# Comparing machine learning methods predicting transcriptome from epigenome with applications to association studies

**DOI:** 10.1186/s13059-026-04131-w

**Published:** 2026-07-13

**Authors:** Fatemeh Behjati Ardakani, Shamim Ashrafiyan, Laura Rumpf, Dennis Hecker, Marcel H. Schulz

**Affiliations:** Institute for Computational Genomic Medicine, Goethe University Frankfurt, Theodor-Stern-Kai 7, 60590 Frankfurt am Main, Hesse Germany

## Abstract

**Background:**

Understanding how epigenome variation contributes to gene expression in disease and development is a fundamental challenge. Regulatory regions show cell type-specific epigenome activity and differ in their location, size, and distance to their target genes, complicating discovery and analysis. Recent machine learning models have been proposed to address these problems by learning functions for the prediction of gene expression from epigenomic data.

**Results:**

Here, we use the large IHEC EpiATLAS dataset to benchmark state-of-the-art linear and nonlinear approaches. We optimize each approach for over 28,000 human genes, providing an inferred regulatory catalog of gene models. In-depth comparison reveals that gene characteristics and the epigenomic complexity of the locus influence the difficulty of predicting the epigenome-to-transcriptome association. The model performance is further evaluated using CRISPRi and eQTL validation data. Based on these models, we conduct histone-acetylation association studies in a systematic way to investigate how epigenetic variation impacts gene expression. The model-based analysis revealed genes and regulatory regions linked to B-cell leukemia in patient data with known disease-related functions.

**Conclusions:**

Our work provides a foundation for applications that link epigenome variation to gene expression in human cells, by benchmarking methods on a per-gene basis, illustrating their use in a disease context and making trained models available to the community.

**Supplementary Information:**

The online version contains supplementary material available at 10.1186/s13059-026-04131-w.

## Background

Epigenetic regulation is central to basic cellular processes, such as transcription and post-transcriptional regulation, and thus important in human development and disease [1–3]. Genome-wide measurements of histone marks via ChIP-seq revealed associations with different elements, including gene bodies and cis-regulatory elements (CREs) [4]. Several histone marks are known to associate with active regulatory regions, e.g., the modifications H3K4me3 and H3K27ac, and are therefore used to locate cell type-specific CREs [5, 6]. It has become routine to generate H3K27ac ChIP-seq datasets for case-control medical cohorts to study epigenome variation in disease and identify CREs and genes that are connected to disease in a cell- or tissue-specific way using histone-acetylome-wide association studies (HAWAS) [7–13]. Thus far, a HAWAS is done by first finding H3K27ac peaks that are associated with the disease in question, then other integrative analyses are performed to link peaks to genes, which are then considered disease-related genes. Despite the availability of large datasets of histone marks from disease cohorts, there are few specific methods for such association studies, although progress has been made for methods that allow to associate epigenome variation with changes in gene expression.

Two main modeling paradigms have emerged for learning associations between the epigenome and gene expression, each addressing different biological questions [14]. The first paradigm builds gene-agnostic models, which treat all genes as equivalent training instances to learn associations between epigenomic signals and gene expression within a single biological sample [15–22]. Because these models leverage the large number of genes in a genome, they do not require many biological samples for training. However, they cannot accurately capture gene-specific regulatory architecture, such as the precise locations of cis-regulatory elements (CREs) and the strength of their influence on gene expression.

The second paradigm builds gene-specific models, where a separate model is trained for each gene using many paired epigenome and gene expression samples [23–25]. This approach captures how epigenetic activity regulates a particular gene across tissues, cell types, or individuals, making it especially relevant for studying disease cohorts and association studies such as HAWAS. However, it requires large datasets and is computationally intensive because tens of thousands of models must be trained. Consequently, previous studies have often relied on simple linear models, although evidence suggests that nonlinear associations between histone marks and gene expression can provide additional predictive power [21, 22].

To identify regulatory sites from gene-specific predictive models, several interpretation strategies have been proposed, among which in silico perturbation (ISP) offers a computationally efficient solution [26, 27]. ISP approximates experimental perturbations by simulating the knockout of individual regulatory regions and evaluating the resulting change in the model’s predicted expression of the target gene. This approach enables direct mechanistic interpretation of the model’s learned dependencies, linking epigenome variation to gene regulation.

**Fig. 1:**
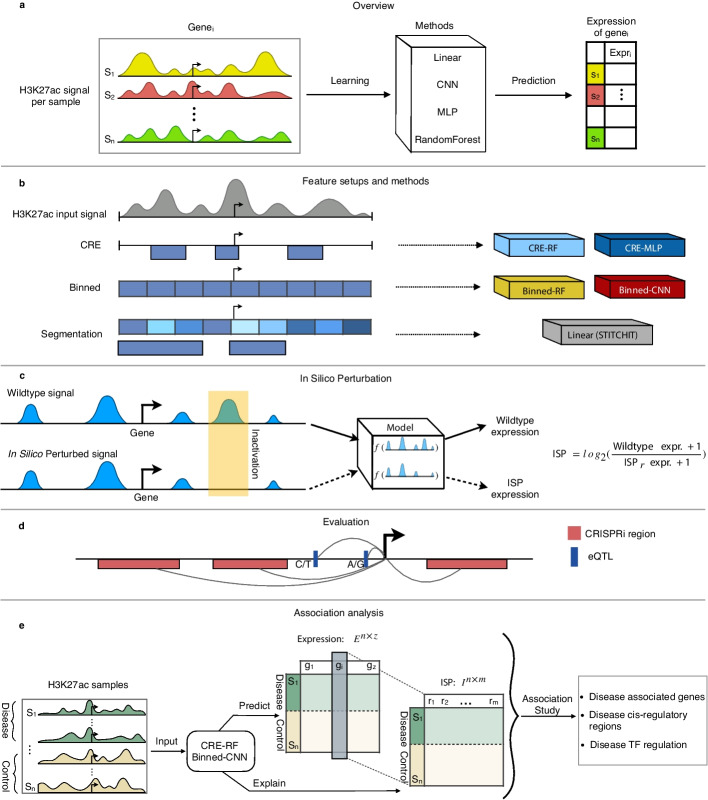
Overview of the model training, feature setups, evaluation of the predicted enhancer-gene interactions and disease-association analysis. **a** Each model is learned per gene to predict gene expression using H3K27ac signal for *n* samples around a gene as input. **b** H3K27ac signal is quantified for different types of feature setups that are combined with machine learning approaches to form five methods for learning. **c** In silico perturbation (ISP) compares the predicted gene expression using wildtype input signal (Wildtype expr), and expression predicted for input signal where one input feature *r* is set to zero (ISP$_r$ expr). **d** Validation is done using external eQTL and CRISPRi data. **e** Overview of the analysis workflow for a histone-acetylome-wide association study using the CRE-RF and Binned-CNN models. Gene expression matrix ($E \in \mathbb {R}^{n \times z}$), where *n* is the number of samples and *z* is the number of genes, is predicted using either method from H3K27ac signal of control and disease samples. Statistical analysis of the predicted gene expression values is used to identify disease-associated genes. Analysis of in silico perturbation values (ISPs) reveals disease-associated regulatory regions, represented by the matrix $I \in \mathbb {R}^{n \times m}$, where *m* is the number of regions. From these regions involved transcription factors (TFs) can be delineated

This work contributes to the field in the following ways (Fig. 1a-e): (i) we compare state-of-the-art machine learning approaches for predicting gene expression from H3K27ac data in a gene-specific manner, (ii) we devise a systematic benchmark on the large EpiATLAS dataset from IHEC [28, 29], (iii) model interpretation and validation, and (iv) inspired by model-based transcriptome-wide association studies [30], we propose a two-step approach for HAWAS. Model interpretation is performed using ISP (Fig. 1c), which systematically quantifies the contribution of individual regulatory regions to gene expression predictions and provides the mechanistic basis for downstream validation and association analyses.

To assess the robustness and biological generalizability of expression prediction, we applied two dataset partitioning strategies with distinct goals to evaluate model performance in different biological contexts. First, a random sample-level 80:20 train–test split assessed predictive accuracy on unseen samples across the full diversity of tissues and cell types. This setup reflects practical use cases and leverages all available regulatory variation to build informative models for downstream analyses. Second, to assess the model’s ability to generalize to previously unseen cellular contexts with distinct regulatory programs, we used a stricter held-out cell type validation, for a subset of genes, excluding entire cell types from training and reserving them for testing.

To translate these predictions into disease-associated regulatory insights, our two-step HAWAS approach (Fig. 1e) integrates predictive modeling and interpretive analysis. The first step reveals genes associated with epigenetic variation in the disease. The second step identifies the regulatory regions linked to disease-relevant genes. We illustrate both steps of HAWAS on data from two groups of patients with chronic lymphocytic leukemia and grade 1 colon adenocarcinoma and suggest novel genes and regions associated with these diseases. CLL is particularly well suited for this analysis because it arises from relatively homogeneous B lymphocytes and exhibits extensive epigenetic dysregulation [31], including alterations in H3K27ac-marked regulatory elements that influence gene expression. The colon adenocarcinoma samples provide a complementary solid tumor context, allowing us to evaluate whether the framework generalizes beyond hematological malignancies. In addition, both cohorts provide matched H3K27ac and RNA-seq measurements, enabling validation of model-derived associations with observed transcriptional changes. To support future studies and data integration efforts, we created the EpiExpress repository, which provides access to the best-performing models for use on additional datasets.

## Results

### Benchmarking of state-of-the-art machine learning methods for expression prediction on unseen samples across known cell types

The application of epigenome-to-expression prediction models relies on having accurate models. To evaluate whether current methodology yields such accurate models, we conducted a comprehensive benchmark study for gene-specific approaches (Fig. 1a). The benchmark dataset is a unique resource comprised of 965 human samples originating from 51 cell types/tissues for which both RNA-seq and H3K27ac ChIP-seq data were available in the IHEC EpiATLAS (https://ihec-epigenomes.org/epiatlas/data/) (Additional file 1: Fig. S1a).

We applied state-of-the-art machine learning approaches to the task of gene-specific learning. Nonlinear associations were accommodated using convolutional neural network (CNN) and multilayer perceptron (MLP) architectures (Additional file 1: Fig. S2), and random forests (RFs). In addition, we used **STITCHIT**, a method that combines signal segmentation and sparse linear regression [25].

We applied various strategies to generate a collection of putative regulatory elements as features for the subsequent model learning step (Fig. 1b). The different feature setups result in varying numbers of input features (Additional file 1: Fig. S1b). We quantified the epigenetic signal in predefined consensus cis-regulatory elements (CREs) identified by the ENCODE project [32] in a 1 MB genomic window as used in previous studies to capture distal cis-regulatory interactions [33, 34], surrounding the most 5’ GENCODE TSS of a gene (**CRE** strategy). The corresponding models are denoted as CRE-MLP and CRE-RF. Additionally, we introduced a **binned** approach, where the features were defined as 10,000 consecutive, non-overlapping bins of 100 base pairs (bp) [16], which gave the models full flexibility to weigh regions of interest. This specific feature configuration was chosen for an RF-based approach (Binned-RF) and for a CNN-based approach referred to as Binned-CNN. The linear **STITCHIT** algorithm incorporates an integrated feature selection approach that starts by dividing the genome into small equal-sized bins, similar to the binned strategy, but successively merges adjacent bins into larger segments. Applying CNNs to the CRE-based or STITCHIT setup is not appropriate, as the CRE regions are not adjacent and are unsuitable for convolutional operations. Thus, in total five different gene-specific machine learning (ML) approaches were compared that differed in their architecture and ability to handle nonlinear associations. To examine whether self-attention improves expression prediction for CRE-MLP, we compared the baseline MLP models with a variant containing a single-head self-attention layer.

To evaluate model performance under complementary biological scenarios, we used two validation strategies. First, a random sample-level 80:20 train–test split was applied as our primary evaluation strategy to assess prediction performance on unseen samples across the full diversity of tissues and cell types represented in the dataset. This setup reflects the practical scenario of predicting gene expression in new samples while incorporating the broad diversity of regulatory information available during training, thereby enabling construction of informative predictive models for downstream interpretation and association analyses. In this context, training across the full range of available cell types is advantageous, as excluding entire cell types may remove biologically informative regulatory variation and reduce predictive performance. Second, to evaluate biological generalizability across a select subset of genes, we performed a held-out cell type validation in which distinct cell types were excluded from training and used exclusively for testing. This setting provides a stricter and unbiased assessment of model generalizability, evaluating the ability to extrapolate to previously unseen cellular contexts with potentially distinct regulatory programs.

### Comparison reveals gene-specific challenges for accurate prediction on unseen samples across known cell types

All subsequent analyses were performed on 28, 180 genes that passed our quality filtering criteria on the EpiATLAS. Parameter optimization was done for each gene and each ML approach separately. For evaluation under the random 80:20 sample-level split, mean squared error (MSE) and the Pearson correlation coefficient between predicted and actual gene expression were calculated on unseen test samples. The corresponding results for the held-out cell types are reported in “Performance on unseen cell types” section.

**Fig. 2:**
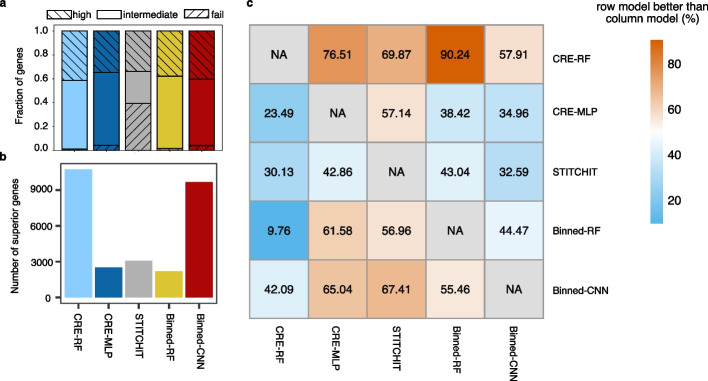
Performance assessment of the five machine learning approaches. **a** Genes are partitioned according to performance (Pearson correlation) into the classes *high* ($\ge 0.7$), *intermediate* ($\ge 0.3$) and *fail* ($<0.3$) for each method. **b** Barplot of the number of genes where a method outperforms all other methods (best Pearson correlation). **c** Heatmap displaying pairwise comparisons between methods, illustrating the percentage of genes for which the method in each row outperforms the method in each column, based on MSE. Correlation and MSE are always estimated on the test set

In order to better characterize the resulting gene models, a partitioning into three quality sets (*high* $\ge 0.7$, *intermediate* $\ge 0.3$ and *fail* $<0.3$) based on test correlation was obtained (Fig. 2a). The *fail* class also included cases for which a method did not produce any model. The methods with the largest fractions of models achieving high correlation values were CRE-RF (41%) and Binned-CNN (40%). The CRE-RF was also the method with the lowest percentage of failed models (1%). Across genes, all models showed large variance in their accuracy to predict unseen samples (Additional file 1: Fig. S3a), highlighting the challenge of learning models per gene. The lowest median error was obtained for CRE-RF (MSE 3.8), closely followed by the Binned-CNN (MSE 3.85). Of all the methods, CRE-MLP had the highest error (median MSE of 4.19). For some genes, some methods failed to obtain a reliable model, which was particularly often the case for STITCHIT due to internal filtering on discretized gene expression (failed for 11,547 genes) (Additional file 1: Fig. S3b). More specifically, STITCHIT requires that at least one percent of each discrete expression value (0 or 1) must be present to ensure sufficient variance in its feature selection procedure.

To assess the impact of an attention mechanism in CRE-MLP, we incorporated an attention layer into the one hidden layer (NN1) and two hidden layers (NN2) topologies and evaluated its effect on model performance. Across all genes, the effect of attention was mixed. For the NN1 topology, attention improved validation loss for some genes but led to poorer performance for others, whereas for the NN2 topology, performance consistently worsened (Additional file 1: Fig. S4a, c). The attention-augmented models also required substantially longer training times (Additional file 1: Fig. S4b, d). For comparison, we selected the best-performing attention model between NN1 and NN2 and compared it to the best-performing baseline MLP. This analysis revealed that the attention models performed worse for more than 60% of the genes based on validation loss and test MSE. (Additional file 1: Fig. S4e-g)

To determine whether any single approach consistently outperformed all other methods, the number of gene models where a given method achieved the highest Pearson correlation was obtained (Fig. 2b). CRE-RF ranked first (11, 404 genes), followed by Binned-CNN (10, 004 genes). However, each method had a subset of genes for which its performance was uniquely superior. For a more detailed analysis, the ML approaches were compared pairwise. Figure 2c shows the percentage of a method (row) outperforming another method (column) based on MSE (other metrics in Additional file 1: Fig. S3c, d). The CRE-based feature strategy outperformed the binned strategy for $90.24\%$ of the genes when comparing the CRE-RF with the Binned-RF, underlining the importance of feature selection in such challenging learning problems. When comparing the two best-performing methods, CRE-RF outperformed Binned-CNN in $57.91\%$ of cases.

**Fig. 3:**
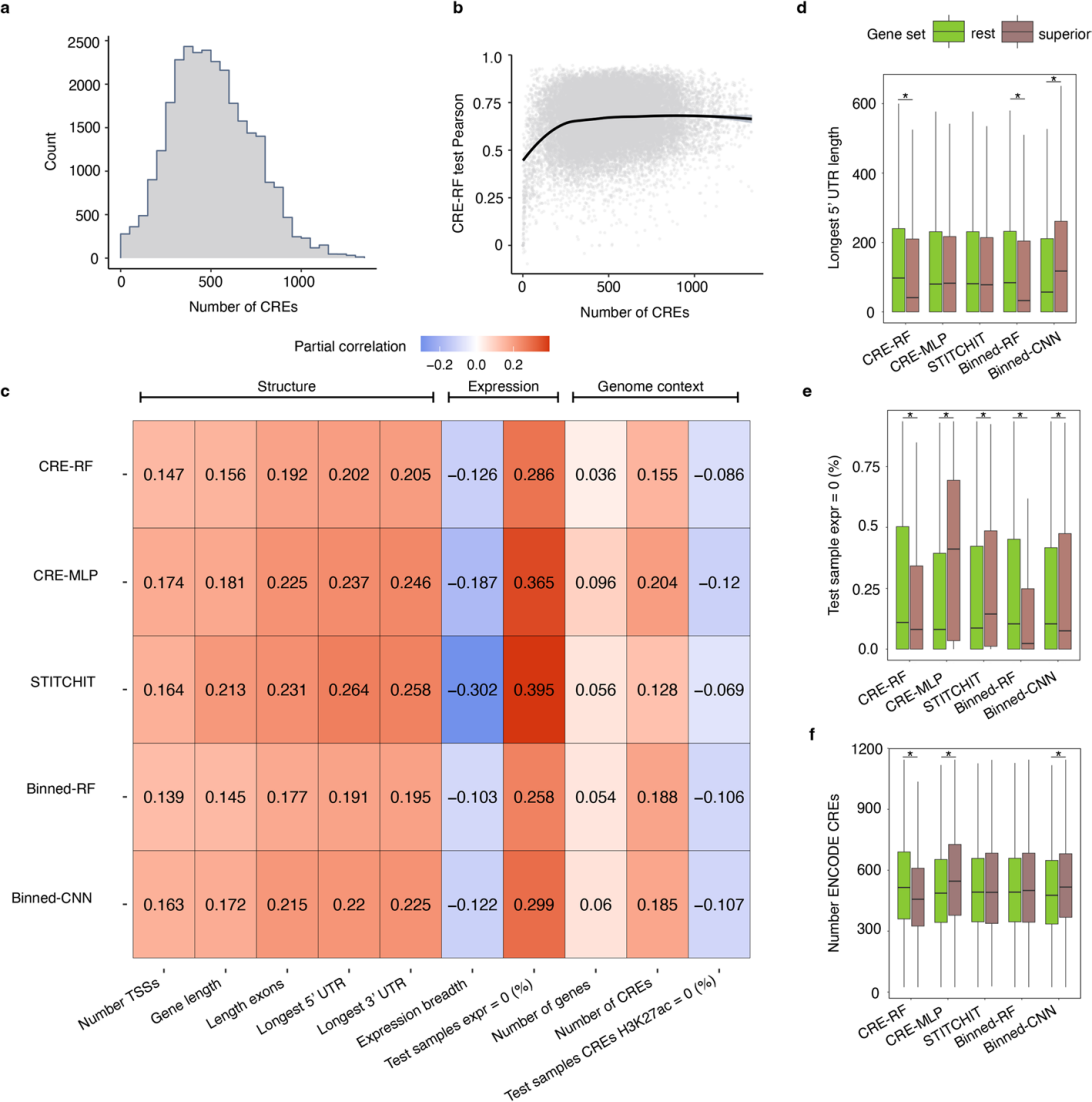
Investigation of gene characteristics that affect model performance. **a** Histogram for the number of gene regulatory regions (CREs) located within a 1 MB gene window. **b** Scatterplot comparing the number of CREs and model performance of the CRE-RF (Pearson correlation). **c** Different gene descriptors were organized into three categories that characterize a gene’s *structure*, *expression* and *genome context* (Additional file 1: Fig. S5a). The impact of one descriptor with the model performance (Pearson correlation) was assessed using partial correlation analyses to correct for confounding correlated descriptors from other categories. The Heatmap shows partial correlation values between each model (row) and descriptors grouped by category (columns). Structure: number of TSSs, gene length, total exon length, lengths of the longest 5’ and 3’ UTR; Expression breadth: fraction of cell types/tissues ($n = 58$) in the entire dataset where the target gene is expressed (TPM $\ge$ 0.5), fraction of test samples with expression $=0$; Genome context: number of other genes, number of CREs in the 1 MB gene window and the fraction of CREs with H3K27ac signal $=0$ in the test samples. **d, e, f** Investigation of genes for which a method has the best MSE value (superior) and all remaining genes (rest) for three descriptors. Boxplot for the length of the longest 5’ UTR (**d**), the fraction of test samples with expression $=0$ and (**e**) the number of CREs in the 1 MB window (**f**). Center line is median, boxlimits correspond to IQR, whiskers to 1.5x IQR. Outliers are not shown. An unpaired Mann-Whitney U Test was performed between the superior and rest gene sets for each gene descriptor and each method separately (*p*-value $\le$ 0.05 indicated with an asterisk). Additional file 1: Fig. S5d-i show the boxplots for the remaining descriptors. Pearson and partial cor- relation values were estimated on the test set

To gain insight into what could explain the variable performance of ML approaches in gene subsets, a number of gene-related descriptors were investigated that may affect training or generalization performance. The descriptors were grouped into three categories related to *structure*, e.g., gene length, gene *expression* variability or *genome context*, e.g., number of CREs in the surrounding area (Additional file 1: Fig. S5a) which showed a large variability between genes (Fig. 3a). Figure 3b illustrates the relationship between one example descriptor (number of CREs) and model performance for CRE-RF, while equivalent analyses were conducted for all other models as well. Two additional example descriptors (exon length and longest 5’ UTR) for CRE-RF are shown in Additional file 1: Fig. S6a, b.

A systematic assessment of all gene descriptors and model accuracy was performed using a partial correlation approach that corrects for performance loss caused by descriptors from other categories (Fig. 3c). All structural descriptors had a positive association with model performance for all approaches. For all methods, the strongest positive influence on model performance was observed for the number of test samples with zero expression. This indicates that all methods could predict tissue-specific genes more easily. Accordingly, the negative impact of the expression breadth shows that it was more difficult for the methods to predict genes that are broadly expressed across all cell types/tissues. The genome context descriptors showed a weak association with model performance. Of all descriptors, the number of surrounding genes had the weakest influence, suggesting that genes in gene-dense regions are not more challenging than those without many other genes in their surrounding. A weak positive association was observed for the number of CREs (Fig. 3b), which was particularly pronounced for up to 300 CREs around a gene. In contrast, we observed a weak negative association with H3K27ac signal sparsity across all models. This pattern indicates that greater sparsity of H3K27ac signals may limit the model’s capacity to capture informative regulatory cues. Thus, it is more difficult to infer gene expression, particularly for genes that remain transcriptionally active despite weak enhancer signatures (Additional file 1: Fig. S5k).

Overall, we observed consistent effects of the gene descriptors across methods, with minor differences in association strength. There was also a dependency of the model performance on the biotype of a gene. Consistently across all models, the expression of protein-coding genes was predicted with a higher accuracy than of non-coding RNA genes or pseudogenes (Additional file 1: Fig. S5b, c). Protein-coding genes displayed characteristic differences in several descriptors including higher number of CREs relative to non-coding RNA genes and pseudogenes, suggesting reasons for the difference in performance (Additional file 1: Fig. S7a-h). Nonetheless, models trained for non-coding RNA genes and pseudogenes were still giving accurate predictions.

Additional insights were gained by investigating the difference in the distribution of gene descriptors of genes for which one method outperforms all others. For example, Fig. 3d shows the length distribution of the longest 5’ UTRs per gene where a method performed strictly better than the other methods. While we observed previously that method performance was positively associated with the 5’ UTR length, Binned-CNN performed particularly well when 5’ UTRs were long in comparison to other methods. The CRE-MLP method was best when many genes had zero expression (Fig. 3e) and many CREs (Fig. 3f), while both RF methods were best for genes with few test samples with zero expression. As RF approaches average over trees it is difficult to predict exactly zero. Overall, ML methods differed in their ability to extract useful information from H3K27ac data on gene expression.

### Model interpretation via in silico perturbation

**Fig. 4:**
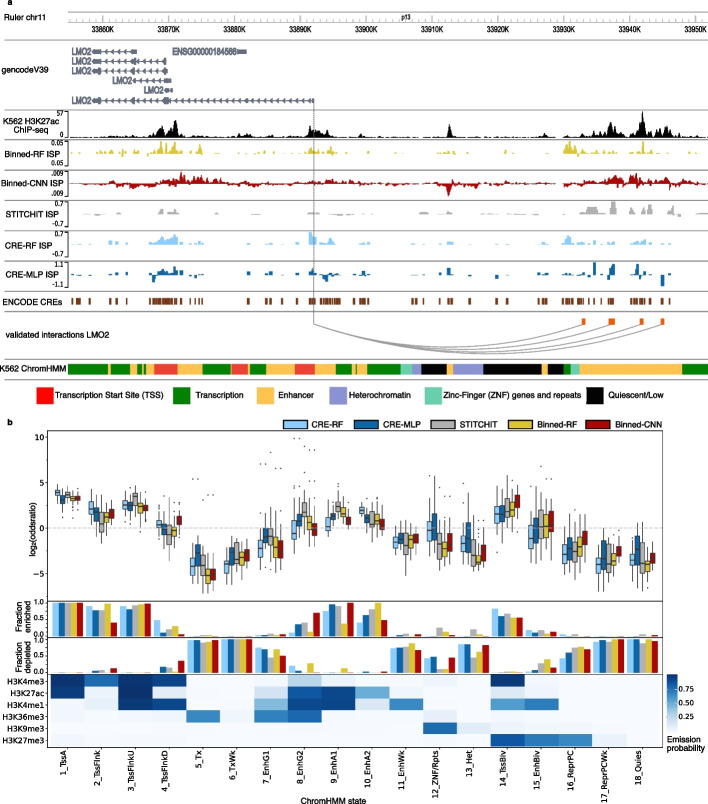
Illustration of the in silico perturbation approach. **a** WashU Browser [35] view of the K562 H3K27ac ChIP-seq signal track at the *LMO2* locus (*top*), followed by the K562 ISP tracks for each method, pre-defined candidate CREs from ENCODE [32], CRISPRi-validated interactions [36] and ChromHMM states [37]. Note that the ISP scale is different for each method, thus not directly comparable. The EpiATLAS K562 sample was used as training data (IHECRE00001887). The ChromHMM states TssA, TssFlnk, TssFlnkU, TssFlnkD were merged together into state *TSS*, Tx and TxWk into *Tx*, and EnhG1, EnhG2, EnhA1, EnhA2, EnhWk into *Enhancer*. **b** Enrichment of ChromHMM states among the top 1,000 regions with the highest absolute ISP. The log$_2$(oddsratio) and the fraction of samples enriched/depleted (*p*-value$~\le ~0.05$) were calculated with a two-sided Fisher’s exact test. Includes all samples and genes with CRISPRi or eQTL data. The emission probabilities of the states were taken from Roadmap Epigenomics Consortium et al. [38]

The key to interpreting the models and revealing underlying regulatory mechanisms lies in the feature importance, meaning which regulatory regions are relevant to predict the expression of a target gene. As a measure of feature importance, an in silico perturbation (ISP) approach (Fig. 1c) was applied. For a target gene, each putative regulatory region was iteratively knocked out, i.e. each feature was set to zero, and the predicted expression value of the pre-trained model was obtained. The ISP was then calculated as log-ratio between wildtype and perturbed expression prediction. To ensure robustness of the ISP scores, we also evaluated perturbations using alternative feature values, which produced consistent results (Additional file 1: Fig. S8). The ISP can be positive or negative, i.e. the ISP indicates not only the strength of the region-gene interaction but also whether the region has an enhancing or repressive effect on the expression of the target gene. The ISP was used as an alternative to other interpretation methods, such as SHAP [39], which require large computational resources and time, especially in our setup with a large number of models and features. Figure 4b displays the ISP tracks of the five approaches at the *LMO2* locus together with K562 H3K27ac ChIP-seq signal, experimentally validated CRE-*LMO2* interactions and ChromHMM regions [37]. The different feature setups allow different levels of resolution. Overall, the methods largely agreed on the locations of putative and validated CREs, but not necessarily on the direction or strength of the regulatory effect. High ISP values overlapped with ChromHMM states that correspond to active transcription and enhancers and were consistent with experimentally validated regulatory regions affecting *LMO2* expression in K562 cells [36].

A systematic analysis of the overlap of regions with high ISP and ChromHMM states confirmed that the models consider regions as important that show histone modifications related to active TSS and active enhancers (Fig. 4c). Transcribed regions, which are defined via presence of H3K36me3 and absence of H3K27ac, as well as repressed and quiescent regions were depleted of high ISP values.

### Model comparison on CRISPRi and eQTL data

**Fig. 5:**
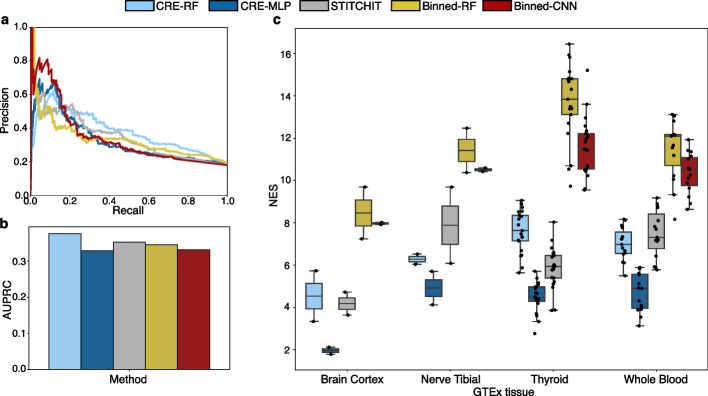
Method comparison using CRISPRi and eQTL data. **a** Precision recall curves of all models based on validated enhancer-gene interactions [36]. An in silico perturbation (ISP) approach was implemented to assess the importance of an enhancer-gene interaction for a model (illustrated in Fig. 1c). Two score calculations were tested: *ISP* and $ISP_{normG}$ (Eqs. 1 and 2). The better one is shown. The curves were created by using the set of interactions for which all models could produce a score (1,099 tested interactions out of which 198 were true positives). Since the interactions were validated in K562 cells, the ISP was calculated for the matching K562 sample from IHEC (HECRE00001887). **b** Barplot with the area under the precision recall curve (AUPRC) for the curves in (**a**). **c** Normalized enrichment score (NES) of the models for enhancer-gene interactions that are supported by eQTL-gene pairs from GTEx [40] calculated with GSEApy (Eq. 3) [41–43]. All enrichment *p*-values are $\le$ 0.05. Shown are the eQTL-gene pairs fine-mapped with DAP-G [44]. The boxplots are formed by the NES of the EpiATLAS samples that were matched to the respective GTEx tissue (Table 1; center line median, boxlimits inter-quartile range, whiskers up to 1.5x inter-quartile range). Similar to (a), *ISP* and $ISP_{normG}$ were tested and the one with higher average NES across all samples was kept. For other fine-mapping methods and score calculations see Additional file 1: Fig. S9

The models were compared with regard to their ability to predict experimentally validated enhancer-gene interactions in K562 cells [36] by estimating the effect of an enhancer (Fig. 1c, d) on a gene’s expression via ISP. The CRE-RF model achieved the highest performance with an area under the precision recall curve (AUPRC) of 0.38, followed by STITCHIT with 0.35 (Fig. 5a, b). The CRE-MLP model yielded the lowest AUPRC with a value of 0.33. For the models trained on the CRE features, normalizing the ISP score per gene increased the performance (Eqs. 1 and 2, Additional file 1: Fig. S9a). Overall, the variation in performance between the models was rather low, which suggests that the regression setup and the ISP approach are more defining for the detection of validated interactions than the specific feature and model setups.

Another source for validation were expression-quantitative trait loci (eQTL) gene interactions, for which IHEC samples were matched to tissues from GTEx (Table 1). When compared on eQTL data, the models with the binned feature setups had the highest enrichment of eQTL-supported enhancer-gene interactions among their top ranked interactions (Fig. 5c). Binned-RF achieved the highest enrichment for 82.5% of all tested samples. Among the CRE-models, CRE-RF had higher enrichment scores than CRE-MLP. For all models the enrichment was higher when the score was not normalized per gene (Additional file 1: Fig. S9b-g). The results for the three tested eQTL fine-mapping methods were highly similar.

**Table 1:** Overview of matched IHEC samples for each of the GTEx eQTL sets. GTEx tissues are matched to EpiATLAS biosamples according to the EpiATLAS metadata file (v1.1). For a mapping of the individual samples see supplementary material IHEC_ValidationSamples.txt

GTEx tissue	EpiATLAS biosample	# samples	EpiATLAS metadata column used
Brain_Cortex	brain	2	harmonized_tissue_type
Nerve_Tibial	tibial nerve	2	harmonized_tissue_type
Thyroid	thyroid gland	21	harmonized_tissue_type
Whole_Blood	venous blood	15	harmonized_sample_ontology_intermediate

**Fig. 6:**
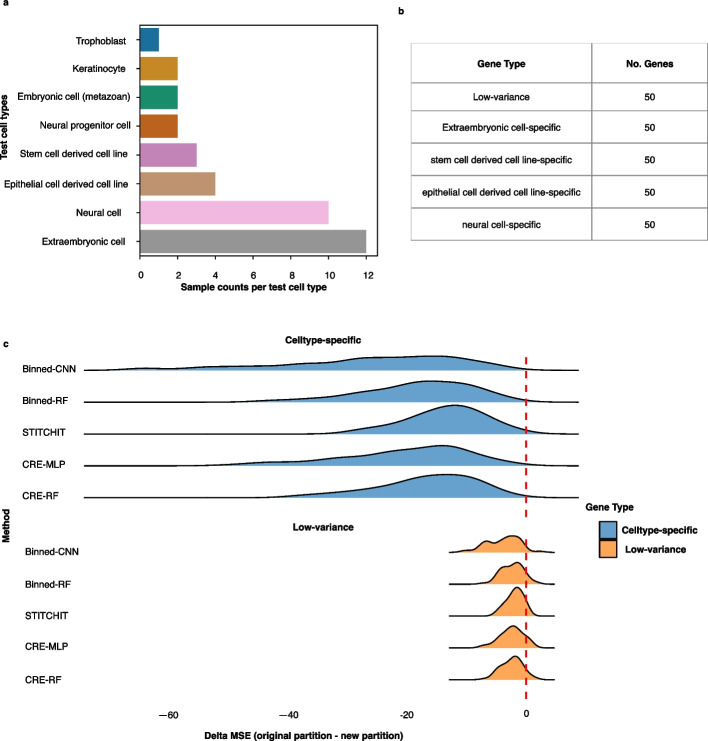
Assessing model generalizability across distinct cell types. **a** Bar plot showing the number of samples available for each selected test cell type used in the cell type-specific partitioning. **b** Table summarizing the number of genes included in each category: low-variance genes and four cell type-specific gene sets corresponding to the test cell types with the largest sample sizes (50 genes per category). **c** Density distribution of the change in prediction error (*Delta*MSE = original partition - cell type-specific partition) across different methods, shown separately for cell type-specific and low-variance genes. Positive *Delta*MSE values show improved performance under the cell type-specific partitioning

**Fig. 7:**
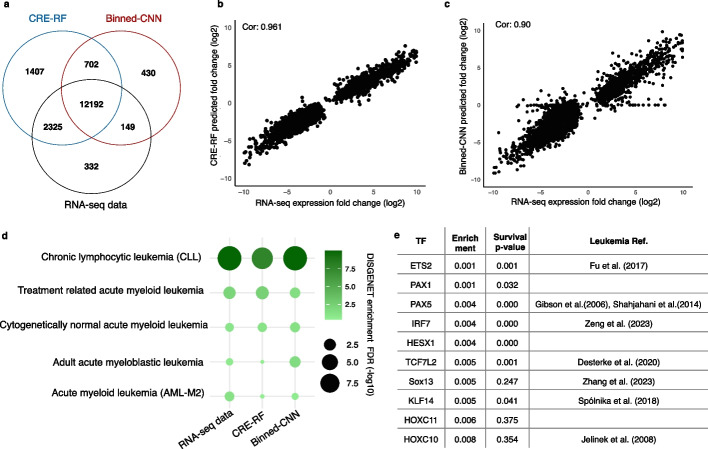
Model-based histone-acetylome-wide association study using pre-trained ML methods reveals insights into leukemia gene regulation. **a** Venn diagram showing the number of significantly associated CLL genes for Binned-CNN, CRE-RF, and experimentally measured RNA-seq data from the same individuals. **b, c** Scatterplot of gene expression fold change values (log2) for real RNA-seq measurements versus predicted for 14,998 genes (differentially expressed in real data) (Pearson correlation indicated in the plot). Comparisons shown for CRE-RF (**b**) and Binned-CNN (**c**) predictions. **d** Dot plot illustrating the overlap of known leukemia genes (DisGeNET database [45]) and CLL genes detected by CRE-RF, Binned-CNN, and RNA-seq expression analysis (enrichment test -log$_{10}$ FDR). **e** Table showing the top 10 enriched transcription factors (TFs) identified by motif enrichment with PASTAA [46] on 49,920 CREs associated with CLL using CRE-RF models. PASTAA enrichment FDR and supporting references are shown in the second and last columns. Survival *p*-value is obtained from Kaplan-Meier survival analysis with log-rank test [47]

### Performance on unseen cell types

So far, model performance was evaluated using random sample-level splits, reflecting prediction on unseen samples. However, this setup does not test generalization to unseen cell types. To address this, we defined a cell type-specific partitioning based on cell type similarity. For this new splitting, the test partition had 36 samples from 8 distinct cell types that were not in the train partition (Fig. 6a and Additional file 1: Fig. S11). To examine the model performance for this partition, we picked 250 informative genes including low-variance and cell type-specific genes. The 200 cell type-specific genes from 4 cell types were unique to cell types in the test partition, which means that the models never saw high expression of these genes during the training. We added 50 low-variance genes, defined as those with the lowest expression variance across all cell types, to represent genes with stable expression (Fig. 6b).

We retrained all models on the cell type-specific partition and evaluated them on the 36 test samples. This experiment directly tests each model’s ability to generalize to previously unseen cell types. As expected, predictive MSE increases compared to the original random partition, but the drop in performance was most pronounced for cell type-specific genes, consistent with their highly context-dependent regulation. In contrast, low-variance genes, which exhibit low variance and broad expression, showed small MSE changes, reflecting their stable regulatory patterns (Fig. 6c). All methods showed higher MSE when evaluated on unseen cell types, suggesting that epigenome to expression prediction models are partially cell type dependent. However, the models still captured meaningful expression variation especially for low-variance gene groups, demonstrating a reasonable degree of generalization beyond the training cell types. These results emphasize that while the models can extrapolate to biologically distinct contexts, full generalization remains challenging when the test cell types differ substantially from those seen during training.

### A novel concept for conducting histone-acetylome-wide association studies

The availability of accurate prediction models enabled a novel application for conducting a histone-acetylome-wide association study (HAWAS) with H3K27ac data for the discovery of disease genes. We suggest a new model-based approach to first find genes associated with the disease and then the relevant H3K27ac signal regions (Fig. 1e). First, a trained ML model is used to predict the expression in control and disease samples for over 28,000 human genes. Then, an association test per gene is performed, to identify genes that show significant alteration in their predicted expression (HAWAS-gene). Second, for these candidate disease genes, the ML model is used to estimate the importance for all gene features by calculating the ISP values. These are used for a second association test to identify the regulatory regions (features) of the ML model where the ISP values differ between control and disease samples (HAWAS-region).

The trained models from the two best-performing methods (CRE-RF, Binned-CNN) were applied to chronic lymphocytic leukemia (CLL) patient data. CLL is known to lead to large differences in epigenomes and transcriptomes of patient cells [31, 48]. Twenty six adult samples, consisting of 12 healthy and 14 CLL samples (including both female and male) were selected. However, three samples were removed as outliers based on PCA analysis, and one sample was excluded due to unknown sex. After filtering, 22 samples (10 healthy and 12 CLL) remained for analysis. After conducting the HAWAS-gene test, different numbers of significant genes were identified for each approach: 13,473 for Binned-CNN and 16,626 for CRE-RF. For comparison, differential analysis of gene expression with measured RNA-seq data from the same biological samples resulted in 14,998 genes and large overlaps with the computational methods (Fig. 7a). The relatively high number of differentially expressed genes is consistent with previous studies reporting widespread transcriptional and epigenetic alterations in CLL, reflecting the extensive molecular reprogramming characteristic of this disease [31]. This suggests that both ML approaches captured relevant biological differences in expression. Strikingly, the CRE-RF based analysis identified 97% of the genes obtained from RNA-seq analysis. Indeed, the predicted fold changes between healthy and control samples were most similar for the CRE-RF (Fig. 7b, $R=0.96$) and less accurate for the Binned-CNN (Fig. 7c, $R=0.90$). Enrichment analysis with known CLL genes (DisGeNET database [45]) indicated a significant overlap for each approach (Fig. 7d), suggesting that the models were successfully identifying genes involved in CLL pathogenesis.

We also compared the HAWAS gene-test to conventional approaches for associating histone modification changes to genes. We took regions with differential signal ($n = 223,586$) and associated them either to the nearest gene ($n = 22,409$) or to their putative target genes according to regulatory interactions predicted with the generalized Activity-by-Contact (gABC) score ($n = 25,370$) [49, 50]. The conventional approaches were less specific and found thousands of genes without a significant expression change. 56% (nearest gene) and 55% (gABC) of the identified genes were differential according to the RNA-seq data, while 87% of the genes found by CRE-RF and 91% of Binned-CNN genes were differentially expressed. As a consequence, genes found by the HAWAS-gene tests had stronger expression changes (Additional file 1: Fig. S12).

Because CRE-RF was the most accurate in predicting differential RNA expression, it was used for the HAWAS-region analysis to uncover 49,920 significant regions from the 16,626 significant genes ($FDR\le 0.05$). 45.1% of the identified regions overlapped sites that were significant in a direct comparison of the ChIP-seq signal between control and disease [49]. Significant variation in active euchromatin may affect the binding of transcription factors (TFs) in those regulatory regions. A TF motif enrichment analysis [46] of the CRE-RF identified regions revealed 30 associated TFs (FDR 0.01). Among the top 10 TFs, 7 were found to be relevant to leukemia according to literature evidence (Fig. 7e) [51–58]. The remaining 20 TFs, 14 of which also show leukemia relevance based on literature, are provided in Additional file 1: Table S1. Kaplan-Meier survival analysis with log-rank tests (median cut point) was conducted for the top 10 TFs between control and CLL samples from established cohorts using the Survival Genie2 tool [47]. Seven out of the ten TFs had a significant survival difference with *p*-value $\le 0.05$ (Fig. 7e and Additional file 1: Fig. S13). Thus, performing the HAWAS on the model predictions provided valuable insights into the underlying genes and regulating TFs in CLL, highlighting the utility of the developed models for biomedical research applications.

To assess the generalization of the model-based HAWAS beyond CLL, we applied the trained CRE-RF and Binned-CNN models to grade 1 colon adenocarcinoma samples (8 healthy, 4 disease). We conducted the HAWAS-gene analysis to identify disease-associated genes based on predicted expression changes. In total, 6,918 significant genes were detected from RNA-seq data, 5,604 from CRE-RF, and 3,989 from Binned-CNN, with substantial overlaps between predicted and measured sets (Additional file 1: Fig. S14a). Predicted and observed $\log _{2}$ fold changes were strongly correlated ($R=0.94$ for CRE-RF; $R=0.80$ for Binned-CNN; Additional file 1: Fig. S14b, c). To validate the gene sets found by our two models, we compared them with known colon cancer genes from DisGeNET (Additional file 1: Fig. S14d). We conducted the HAWAS-region analysis for CRE-RF to find significant regions and related TFs. Two TFs were identified as significant, both of which have been reported in the literature to be associated with colon cancer(Additional file 1: Fig. S14e). These results indicate that the models capture relevant expression changes even in diseases with diverse origin and tissue types, supporting the robustness of the HAWAS framework across conditions.

## Discussion

The EpiATLAS resource was used to train and benchmark various machine learning approaches in a gene-specific learning setup. A challenge for the comparison was that a method required individual optimization for over 28,000 genes. Naturally, there was a limitation in the variety of model topologies and parameter setups that could be explored in this study. We realized that the feature setup played a crucial role for model performance. The advantage of CRE-RF, with predefined regions, over the Binned-RF could originate from the removal of many irrelevant features before learning. Using regularization may be an alternative way to improve performance of the RF-based approaches [59]. Further, there are many additional topologies and components for neural networks that one could try. We expect that using the EpiATLAS dataset further improvements will be made in the future. Also, it is likely that improvements can be made by adjusting for gene descriptors that affect a model’s performance (Fig. 3).

To understand how different model structures extract the relationship between H3K27ac and gene expression, we compared their architectural assumptions. From a structural perspective, the strong performance of CRE-RF and Binned-CNN arises from how each model captures regulatory information. The CRE-RF uses biologically defined cis-regulatory elements as input features, reducing noise and allowing the random forest to learn nonlinear interactions among relevant regions efficiently. In contrast, the Binned-CNN leverages the spatial organization of the chromatin signal, where convolutional filters detect local enhancer patterns and position-independent features across the genomic window. These design choices enable CRE-RF to benefit from biologically constrained input, while Binned-CNN captures spatial dependencies in the epigenetic landscape. CRE-MLP shares the CRE-based representation with CRE-RF, but its fully connected layers must integrate a larger set of CRE inputs without explicit spatial or biological constraints, which may contribute to its weaker and less stable performance. STITCHIT segments the binned H3K27ac signal into putative regulatory units based on expression correlation and then predicts expression from these segments, making it sensitive to genes with clear H3K27ac expression coupling and unable to provide models for all genes. Binned-RF uses the same 10,000 bins representation as Binned-CNN but, like CRE-RF, treats bins independently and therefore captures only local effects without exploiting spatial continuity. Together, these differences in input representation and architectural bias explain the overall performance ranking of the methods and their similar, yet not identical, dependencies on gene level descriptors. It is reasonable to assume that expression prediction for a gene that shows a complex regulatory pattern (many CREs, nonlinear combinations) is difficult at the current size of 792 training samples. To improve convergence for CNN models, we used a warm-start initialization procedure, which increased accuracy for many genes, but applying a similar strategy was not feasible for MLP models, as their feature space differed between genes.

We observed that the CRE-MLP performs better for genes with a high proportion of zero expression test samples (Fig. 3e). The lower MSE of the CRE-MLP for these genes mainly reflects its ability to accurately predict near zero expression values, rather than improved modeling of complex regulation. The CRE-MLP’s ReLU based architecture naturally produces small output values in response to such low inputs, resulting in predictions that closely match the observed near zero expression levels. Consequently, the model achieves low absolute errors for these genes.

While Transformer-based and attention-augmented architectures have achieved notable success in sequential and natural language applications, our results indicate that they provide limited benefit in the current setting. 792 training samples per gene may not be sufficient to effectively train attention layers with additional parameters. In our experiments, integrating a one-head self-attention layer before the MLP did not improve performance for most genes and substantially increased training time (Supp Fig. S4). Nonetheless, more complex Transformer-based extensions could be explored in future work, particularly if larger multi-tissue datasets become available.

A potential way to further improve prediction performance is to borrow information across genes by training a unified model over all $\sim$28,000 genes and the whole genome. A prior study using CpG methylation values [60] has shown that such genome-wide frameworks can predict expression very accurately. Applied to our setup, this approach would require handling a very large feature space (one million CRE features or several million binned features), but the resulting model may benefit from learning global chromatin expression relationships. An alternative approach is to group genes for model training based on predefined similarity criteria, for example, cell type specificity. Exploring such genome-level modeling is an open research direction.

To evaluate the generalizability of our models in a held-out cell type setting, we implemented a cell type-specific partitioning strategy in which a subset of distinct samples was withheld for testing. Our findings suggest that the observed differences in generalization may stem from intrinsic data heterogeneity and limited representation of rare transcriptional patterns within the training set. The inability of the models to accurately extrapolate to highly distinct cell types highlights a key limitation in cell type–agnostic modeling approaches, where the learned relationships may not fully capture cell type–specific regulatory mechanisms. This emphasizes the importance of including a broader diversity of cell types during training or developing adaptive frameworks that can better transfer learned patterns across cellular contexts. Furthermore, the relatively stable performance on low-variance genes implies that baseline expression levels are more easily captured by the models, while dynamic or context-dependent genes remain difficult to predict.

Methods from explainable AI [39, 61] are commonly used to investigate the contribution of individual features to a model’s prediction. Since over 28,000 gene-specific models were analyzed, we employed the computationally efficient ISP approach to assess how individual genomic regions influence expression predictions. Applying explainable AI methods such as SHAP was computationally infeasible given the scale of our analysis and the substantial runtime associated with model-specific feature calculations. Moreover, SHAP-based inferences can sometimes be unreliable when applied to highly correlated genomic features, as the underlying feature independence assumption may not hold [62]. The ISP method instead quantifies the effect of specific genomic regions by perturbing one region at a time, allowing for direct and interpretable assessment of local feature contributions. However, this design also means that ISP does not capture potential co-regulatory or interactive effects among genomic regions. We acknowledge this as a limitation of our current framework and consider it an important direction for future methodological refinement.

New model-based applications were suggested in the context of HAWAS that estimate genes or regulatory regions of genes associated with a disease. In particular, the approach for model-based transcriptome-wide association studies (TWAS) [30, 63] is conceptually similar, except that in a TWAS disease genes are predicted from genomic variant data, not from epigenome data. Although transcriptomic data are more widely available than histone modification profiles, the motivation for HAWAS is fundamentally different from the data availability motivation behind TWAS. TWAS relies on genotype expression relationships that are constrained by the modest heritability of gene expression, limiting predictive power for many genes. In contrast, histone modifications capture cell type-specific and context-dependent regulatory activity that explains substantially more expression variance and reflects upstream regulatory mechanisms rather than downstream transcriptional states [15]. As a result, epigenome to expression models provide mechanistic insight into enhancer and promoter dysregulation that cannot be obtained from RNA-seq alone. HAWAS therefore complements TWAS by identifying disease-associated regulatory elements even for genes with low genetic heritability, offering a regulatory locus level association framework that is not accessible through genotype-only or expression-only analyses.

Applying the model-based HAWAS to data from CLL and colon adenocarcinoma patients revealed good agreement with RNA-seq differential gene expression analysis and identified regulators associated to survival, even at a relatively small cohort size of 22 leukemia samples (Fig. 7) and 12 colon samples (Additional file 1: Fig. S14). Thus, for future HAWAS the experimental efforts could be limited to measuring only the epigenome, which may be important in situations where measuring RNA transcripts is complicated or may allow lowering sequencing costs. However, it can be expected that the application of the models on cell types that were not part of the EpiATLAS will show reduced performance, particularly for cell type-specific genes.

Although we incorporated a comprehensive atlas encompassing multiple cell and tissue types to train our models, the models remain confined to within-dataset explanations, and any interpretation beyond this should be made with caution. Currently, our models are available for over 28,000 human genes and larger data sets are needed to learn models for genes that had too little variation in the training set. The ability to obtain disease genes and their CREs using case-control epigenome data as illustrated here, will fuel future association studies and reveal cell type-specific roles of the epigenome in disease.

## Conclusions

This study shows that gene-specific machine learning models can learn the epigenetic landscape defined by H3K27ac and its relationship to gene expression. By systematically comparing different input representations and model architectures, we demonstrate that both feature design and model structure are critical for predictive performance, interpretability, and the ability to capture regulatory mechanisms. Biologically informed features and architectures that reflect spatial or regulatory constraints provide clear advantages in modeling these relationships. Importantly, we illustrate the application of these models in a HAWAS framework, enabling the identification of disease-associated genes and regulatory elements directly from epigenomic data. This highlights the potential of epigenome-based modeling not only for prediction but also for regulatory inference, offering a complementary approach to existing association methods and supporting future studies of gene regulation in disease.

## Methods

### EpiATLAS epigenome and transcriptome data

Model training was based on the data of the International Human Epigenome Consortium (IHEC), available via the EpiATLAS portal (https://ihec-epigenomes.org/epiatlas/data/) [28]. The activity of genomic regions was assessed by the H3K27ac ChIP-seq signal, more precisely the average fold-change signal over the background in a given region. For gene expression the expected counts from RSEM [64] were used after size factor normalization with DESeq2 [65]. All 965 samples were included that had both H3K27ac ChIP-seq and RNA-seq available, according to the metadata version 1.1. We provide a mapping of the unique identifiers for the RNA-seq and ChIP-seq experiments on our Zenodo. As cell type/tissue label for the samples, the column *harmonized_sample_ontology_intermediate* was used, which encompasses 51 different ontology terms for the 965 samples. If multiple RNA-seq experiments were provided per sample, the total RNA-seq experiment was chosen. All data is in human genome version GRCh38. As gene annotation we used v38 from GENCODE [66].

### Dataset filtering and preparation

We filtered for genes with $\ge ~2$ expression variance and 90% non-zero values across samples in the RNA data. This resulted in a set of 28,180 genes that we retained for the analyses throughout this study. Additional file 1: Fig. S5b provides details on the biotypes of the retained genes. For all methods, the feature and response matrix were $log_2$-transformed with a pseudo-count of 1. If additional scaling was performed, it was first applied to the training data, and the scaling factors from the train data then utilized to normalize the test data to prevent information leakage. Details on the scaling procedure used by each method can be found in the corresponding sections.

### Feature setups

Since models do not have prior knowledge of where regulatory information is located, three distinct strategies were employed to define the candidate regulatory regions for a gene: *CRE*, *binned*, and *segmentation*. For the *CRE* strategy, candidate CREs from ENCODE were used to identify putative regulatory regions within the 1 MB window surrounding the TSS. More precisely, the candidate enhancers and candidate promoters were downloaded from ENCODE’s SCREEN web interface (v3) [32] and merged into a joint set of 988,415 candidate CREs. This set of CREs was kept fixed for all samples, ensuring a unified feature definition across the dataset. The *binned* setup provides an unbiased spatial representation and for this approach, the abundance of H3K27ac read counts was calculated in consecutive 100 bp bins spanning a 1 MB window symmetrically centered on the 5’ TSS of the gene. Similar to the binned approach, for segmentation-based feature selection the genome was divided into equal-sized bins holding the mean H3K27ac signal in a 1 MB window around the TSS of a target gene. The bins of size 10 bp were successively merged into larger segments within the STITCHIT algorithm. Figure 1b schematically depicts the three feature designs utilized in this study.

### Data partitioning and cross validation for training

To benchmark the performance of the different approaches, the dataset was randomly partitioned into a training ($80\%$) and test set ($20\%$). All reported model performances were calculated on the test partition. For all methods except RF, a cross-validation (CV) procedure was applied for parameter-optimization on the training data. The best model was selected according to the minimum CV-error. The CV-error corresponds to the average mean-squared error (MSE) over the CV-folds. Details on the CV-parameters can be found in the respective method section.

### Setup for machine learning models

**CRE-RF**

Random Forest model was trained using the randomForest function from randomForest library version (4.7.1.1) in R. The data was normalized to the range [0,1] using min-max scaling. Preliminary checks showed that varying key parameters (number of trees, mtry, node size) did not yield meaningful performance improvements, so the defaults were retained:
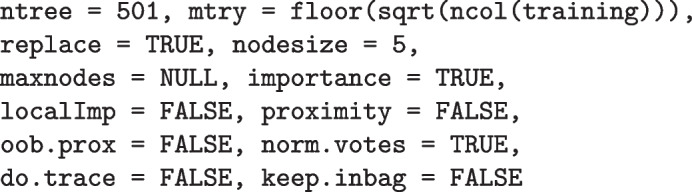


**CRE-MLP**

Two different designs were explored for the MLP models: one with a single hidden layer and another with two hidden layers (Additional file 1: Fig. S2a, b). The single hidden layer MLP consisted of 100 nodes, while the two hidden layer MLP comprised 100 nodes in the first layer and 50 nodes in the second layer. ReLU activation functions were used for all hidden layers, while a linear activation function was applied to the output layer. Hyperparameter tuning was conducted for each topology to identify the most effective model configurations. Given the high computational cost of exhaustive hyperparameter search, parameter combinations were evaluated on a subset of 500 genes. For these genes, we tested variations around the default MLP configuration to assess sensitivity to key parameters. Hidden layer sizes proportional to the input dimensionality ($\tfrac{1}{3}$, $\tfrac{2}{3}$, and $1 \times$ the number of features) were examined, but larger architectures substantially increased training time with minimal performance benefit. Therefore, fixed hidden sizes (100 for the one-layer MLP and 100/50 for the two-layer MLP) were adopted. For the Adam optimizer, the search grid included three learning rates (0.1, 0.01, and the default 0.001). The best-performing rate was 0.001 in approximately 80% of cases, hence it was chosen. Dropout rates of 0.2 and 0.4 yielded similar regularization performance and were both retained in the grid. Because initial randomization had a noticeable impact on model performance, we evaluated (30 random seeds) in our hyperparameter grid. Hence, dropout rates (0.2 and 0.4 for both the first and second hidden layers), batch size (fixed at 32), maximum epochs (set to 1200 with an early stopping criterion of 30 epochs patience), and default learning rates for Adam optimizer (at 0.001) were defined [67]. The data was normalized to the range [0,1] using min-max scaling, then MLP models were trained using the Keras library in R with the help of its built-in validation features. The validation set accounted for 30% of the training data and was used to assess model performance during training. For each gene, the best-performing single-hidden-layer (NN1) and two-hidden-layer (NN2) MLP models were identified based on validation loss, and the final architecture was selected by directly comparing their validation performance (Additional file 1: Fig. S2d) To assess the potential benefit of attention mechanisms, we extended the MLP architecture by adding a single-head self-attention layer preceding the first fully connected layer. The attention mechanism was implemented with standard scaled dot-product attention, applied to the normalized input feature representation before being passed into the MLP. The same training and validation setup as for the baseline MLP models was used to ensure comparability.

**Binned-RF**

We used the random forest for regression on the binned data by utilizing the RandomForestRegressor function from the scikit-learn library in Python. The response was min-max scaled to the range [0,1]. Preliminary checks showed that varying key parameters did not yield meaningful performance improvements, so the defaults were retained:
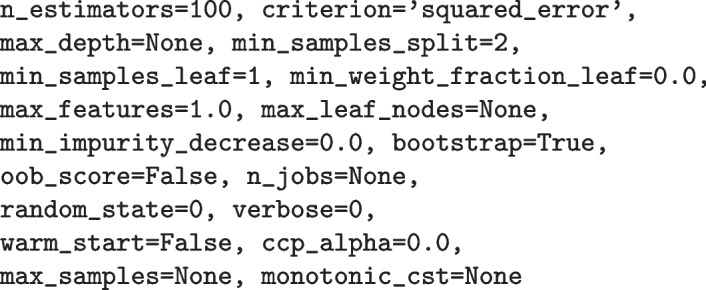


**Binned-CNN**

Given the nature of the features in the binned setup, a convolutional neural network (CNN) was selected. Two primary CNN topologies were employed in this study: the contracting kernel and the expanding kernel (Additional file 1: Fig. S2c, d). In the contracting kernel, the kernel sizes gradually decrease from the input layer to the output layer. Conversely, the expanding kernel topology features progressively larger kernel sizes. For both topologies, a dense layer with 5 hidden nodes connects the convolutional block to the output regression node. We evaluated two learning rates for the CNN models, 0.01 and 0.001. Training with the higher learning rate (0.01) resulted in unstable optimization behavior, with models frequently converging to near-constant outputs and failing to capture variability in gene expression. In contrast, the lower learning rate (0.001) enabled more stable weight updates and led to improved predictive performance. We further assessed the impact of stride length through a grid search over values of 1, 2, and 5. As stride length inversely affects model complexity by controlling the number of trainable parameters, smaller strides increased model capacity. Our experiments demonstrated that a stride length of 5 achieved the most favorable balance between model complexity and effective feature summarization. Additionally, we examined batch sizes of 8, 16, 32, and 64. A batch size of 32 was selected, as it offered an optimal compromise between computational efficiency and model performance. For all models, we tested the CNN results by varying the activation functions in the first and last layers. Specifically, we explored the influence of ReLU and sigmoid activation functions in these layers. Since the initialization of weights was crucial for optimization, a heuristic warm start strategy was developed to initialize the weights (Additional file 1: Fig. S2c). Inspired by the concept of transfer learning, three distinct gene pools were created, each serving as a separate dataset. Specifically, batches of 1,000 genes were randomly selected from the entire gene set, along with their corresponding biological samples, to form one of three datasets. The contracting and expanding kernel topologies were trained on these three datasets using the ReLU activation function throughout. Each gave an optimal model for the 1,000 gene set and the corresponding parameters of each of these three models were used as initial weights (warm start) to train the CNN models in a gene-specific manner. For genes located near the chromosome borders, where a complete 10,000-bin setup was not feasible, we could not transfer the parameter initializations and we thus trained the CNN models using a cold start. Before model training, the response was min-max scaled to the range [0,1]. The features were scaled to unit-variance using a batch normalization layer. All CNN implementations were carried out using the *Keras* library in Python, with a validation split of 0.2 and a learning rate of 0.001. The models were trained using the Adam optimizer for up to 1000 epochs [67], with batch size of 32 and early stopping applied to prevent overfitting (patience parameter set to 5). For both contracting and expanding CNN models, the stride in the first convolutional layer was set to 5. In the expanding model, subsequent layers used a stride of 2, while in the contracting model, a stride of 1 was applied to all remaining convolutional layers. All other parameters were kept at their default settings. For each gene, the best performing contracting-kernel and expanding-kernel CNN models were identified based on validation loss, and the final topology was selected by directly comparing their validation performance (Additional file 1: Fig. S2e).

### STITCHIT

STITCHIT incorporates the identification of putative regulatory elements in a segmentation step before applying model learning. The initial bins were merged into larger segments such that the H3K27ac signal variance within a segment is low across all samples, with respect to discrete gene expression labels. The gene expression labels comprised binary classes $\{0,1\}$ that represent expression and no expression generated by a sample-wise discretization approach described in [68]. The segments that were associated with changes in the continuous expression of the target gene were chosen for the subsequent linear learning step. For this purpose, the Pearson correlation was calculated between the H3K27ac signal and the expression of the target gene across all samples. All segments that passed a significance threshold of $P~\le ~0.05$ were selected as features for predicting gene expression using an elastic net model. A six-fold nested CV-procedure was employed for regularization parameter tuning and model selection on the standardized training data. Details of the STITCHIT algorithm and effects of parameter tuning are available in [25].

### In silico perturbation score calculation

As an estimate for how important a genomic region is for the gene expression prediction in a sample, we calculated an in silico perturbation score (ISP). To do so, the signal of a given region *r* was set to zero in the input matrix and the expression predicted with this perturbed input. The ISP score is then defined as the $log_{2}$-ratio of the predicted expression on the unperturbed data ($Wildtype\ expr$) over the perturbed expression prediction ($ISP{_r} expr$):

1 $$\begin{aligned} ISP_{r}=log_2\left( \frac{Wildtype\ expr +1}{ISP_r\ expr +1}\right) . \end{aligned}$$

Since the models use different genomic regions as features, the regions that are perturbed for the ISP also differ. For the CRE-based models the individual CREs were set to zero and for STITCHIT the putative regulatory elements that it defines per gene were perturbed. Due to the large feature space of the binned models, multiple adjacent bins were perturbed together. More precisely, for a genomic region of interest, bins overlapping this region were set to zero, as well as the neighboring bins for a minimum of 10 bins. To summarize, the score can be calculated for each input feature of each gene model and each sample.

To assess the stability of the ISP score definition, we also evaluated whether the score depends on the perturbation magnitude applied to each feature. Instead of setting the signal of a region to zero, we repeated the perturbation by scaling the feature values to small non-zero levels (0.01 and 0.1) and recalculated the ISP scores for all models. The resulting ISP profiles were highly correlated across all perturbation levels (median Pearson correlation > 0.95; Additional file 1: Fig. S8a), confirming that the small deviations in the perturbation strength does not affect the inferred ISP scores significantly. This demonstrates that the ISP approach provides a stable measure of regional importance independent of the precise perturbation amplitude.

In addition, a score normalized per gene was tested for each model, which was calculated as follows:

2 $$\begin{aligned} normISP_{r}=\frac{ISP_{r}}{\sum _{i \in R}ISP_{i}}, \end{aligned}$$

where *R* denotes all features of the gene. Thus, $normISP_{r}$ sets the effect that the removal of *r* has on the gene expression prediction in relation to the effect of removing any other feature of the gene. For models with a binned feature setup, this approach was not feasible due to the large feature space. This is why for the binned models, all candidate enhancers derived via thresholding their feature importance were taken as *R* (noted as feature fusion).

### Feature fusion

Given that the global feature set for the Binned-CNN models spans the entire 1 MB genomic region divided into consecutive bins, an ad hoc procedure was implemented to derive ISP for these models. This approach included the following steps:

1. **Feature Importance Derivation:** random forest (RF) models were used to determine global feature importance on a per-gene basis.
2. **Binarization via Otsu’s Thresholding:** Feature importance values were binarized using Otsu’s thresholding method, categorizing bins as “active” (1) or “inactive” (0). The *threshold_otsu* function from Python’s *skimage.filters* package was used to calculate Otsu’s threshold.
3. **Fusion of Adjacent Bins:** Consecutive active bins were fused if separated by no more than five inactive bins.
4. **Expansion Strategy:** To ensure adequate impact on model prediction following ISP, fused regions shorter than 1 kb were expanded to a 1 kb span. This expansion sometimes resulted in merging adjacent fused regions, creating larger contiguous regions.
5. **Signal Nullification in Fused Regions:** For each expanded region, the signal in corresponding bins was nullified to calculate the effect of the perturbation on predicted gene expression.
6. **Prediction Ratio Calculation:** The ratio of the original prediction to the ISP prediction was calculated as per the formula outlined in Eq. (2).

Additional file 1: Fig. S10a provides a visual summary of this procedure.

### Enrichment of ChromHMM states among regions with high ISP

To calculate enrichment of ChromHMM states among regions with high ISP (Eq. 1), the regions/features were ranked per model and sample based on the highest absolute ISP across genes. Each region was then assigned to the ChromHMM state with the highest overlap from the ChromHMM annotation of the matching sample. A two-sided Fisher’s exact test was then used to assess whether regions assigned to a certain state occur more often among the top 1,000 regions with the highest absolute ISP than among all other regions. The analysis was limited to samples and genes for which ISP was calculated for the overlap with CRISPRi and eQTL data (see below). The emission probabilities were downloaded from https://egg2.wustl.edu/roadmap/data/byFileType/chromhmmSegmentations/ChmmModels/core_K27ac/jointModel/final/emissions_18_core_K27ac.txt in August 2025 [38].

### Comparison on CRISPRi data via in silico perturbation

As experimentally validated enhancer-gene interactions, the data of Gschwind et al. [36] was used, which is a reanalyzed collection of three CRISPRi-screens in K562 cells [69–71]. More precisely, the file EPCrisprBenchmark_ensemble_data_GRCh38.tsv.gz from their GitHub repository was taken, and all interactions that were labeled as significant in the *Significant* column of the table were considered true interactions. Repressive interactions, where the gene expression increased after enhancer perturbation, were not excluded. Additionally, the interactions were filtered for those with a distance between enhancer and the gene’s most 5’ TSS of $\le$ 500 kb, matching the gene window in our setup. Mapping gene names to Ensembl IDs was done with the API of MyGene.info [72–74]. The resulting set of enhancer-gene interactions is available as supplementary material JointValidatedInteractions_hg38_500kb.txt on Zenodo. Processing of genomic coordinates was done with pybedtools (v0.9.0) [75, 76].

Since all CRISPRi data come from K562 cells, the ISP was done in the EpiATLAS K562 sample (IHECRE00001887). The ISP score was calculated for the genomic regions that are used as input for the models that overlap the validated CRISPRi regions (Eq. 1). Due to the different feature setups, the different models can produce ISP scores for different subsets of validated interactions. The models with a binned feature setup can score all validated interactions, while the CRE-based models and STITCHIT can only generate scores for interactions that overlap their features. The ISP score that is normalized per gene (Eq. 2) was tested as well.

All ISP scores were converted to their absolute values, since the validated interactions contain repressive and activating interactions. To construct PR curves for each model, a score cutoff was increased from the lowest to the highest absolute score in 10,000 steps. All interactions with an absolute score above the cutoff were considered as predicted positive interactions, and all below the cutoff as predicted negatives. Precision and recall were calculated for each cutoff based on the CRISPRi-validated interactions. If multiple features overlapped one CRISPRi-perturbed region, the absolute score of the features was summed.

### Ranking of eQTL-gene pairs

To compare how well the models recover interactions supported by eQTL-gene pairs, tissues from GTEx were mapped to matching samples from the EpiATLAS. Four tissues from GTEx were selected, two with a high number of matching IHEC EpiATLAS samples and two with only two matching samples (Table 1). Data of the three fine-mapping methods available on GTEx (dbGaP accession number phs000424.v8.p2) [40]) were used by accessing the following files: ‘CAVIAR_Results_v8_GTEx_LD_HighConfidentVariants.gz’ for CAVIAR [77], ‘GTEx_v8_finemapping_CaVEMaN.txt.gz’ for CaVEMaN [78] and ‘GTEx_v8_finemapping_DAPG.CS95.txt.gz’ for DAP-G [44]. For each of the models and each of the EpiATLAS samples, a normalized enrichment score was calculated to assess whether interactions supported by eQTL data were ranked higher than those not supported by eQTL data. This was repeated for each fine-mapping method. Each possible enhancer-gene pair for all genes with at least one eQTL in a tissue was scored, meaning each input feature of a model was iteratively perturbed to calculate the ISP for a given sample (Eqs. 1 and 2). For the models with the binned feature setup, the candidate enhancers were defined by thresholding the feature importance of the bins. The absolute scores were subsequently sorted in descending order. To consider the same number of interactions across all methods, the 100,000 highest ranked interactions were taken for each EpiATLAS sample and each model. To also have the same number of interactions supported by eQTL-gene pairs for each method, the set of overlapping eQTL-gene pairs were reduced to the same size per sample (minimum of overlap across methods). Based on the 100,000 interactions, a normalized enrichment score was calculated with the prerank function of GSEApy (v.1.1.2) [43], which is a Python implementation of GSEA [41, 42]. GSEA first derives an enrichment score, which is calculated by going through a ranked list while iteratively updating a running sum statistic that increases if an entry is considered to be a hit and decreases if it is not. In our application this means that the running sum increases if an interaction at a given rank is supported by an eQTL-gene pair and decreases otherwise. The normalized enrichment score is then the ratio of the enrichment score and the average enrichment score across random dataset permutations *R*:

3 $$\begin{aligned} NES=\frac{ES~on~original~dataset}{\frac{1}{|R|} * \sum \limits _{r \in R} ES~on~permuted~dataset}. \end{aligned}$$

GSEA limits the permutation runs to those where the sign of the ES matches to the one of the unpermuted data. The NES gets the same sign as the ES from the original dataset. The number of permutations was set to 100. The weighting parameter p was set to zero.

### Partial correlation analysis

To assess the relationship between gene-specific attributes and the models performance, a partial correlation analysis was conducted across multivariate input arguments using the psych package [79], specifically employing the partial.r function in R. In this analysis, descriptors were organized into three categories: The *structural* descriptors correspond to: i) the number of TSSs, ii) the gene length including introns (gene length), iii) gene length excluding introns (exon length) and the lengths of the longest iv) 5’ and v) 3’ UTRs. The *expression* group comprises i) the proportion of samples in the test set in which the expression of the target gene is nonzero and ii) the expression breadth.

For the expression breadth, all RNA-seq samples from the IHEC EpiATLAS (including those without matching H3K27ac CHiP-seq data) were used and the expression averaged across all samples belonging to a cell type/tissue (|*C*| = 58). To then get an estimate of how broadly a gene is expressed, the fraction of cell types/tissues in which the average expression $\mu _{g,c}$ exceeds 0.5 TPM was taken:

4 $$\begin{aligned} expression\ breadth_{g}=\frac{1}{|C|} * \sum \limits _{c \in C} x \left\{ \begin{array}{ll} x=1,\ if\ \mu _{g,c} \ge 0.5 \\ x=0,\ \text {otherwise} \end{array}\right. . \end{aligned}$$

The *genome context* descriptor set holds: i) the number of ENCODE CREs, ii) the number of genes excluding the target gene and iii) fraction of CREs with zero H3K27ac signal across test samples. All three descriptors were counted within a 1 MB window around the target gene. All descriptors were obtained from the GENCODE v38 annotation file [66].

The partial correlation values between model performance and each descriptor were calculated while conditioning on the other descriptor categories in order to compare the contribution of each descriptor within its respective group (Additional file 1: Fig. S5a). For example, for number of TSSs the correlation was calculated as follows:

5 $$\begin{aligned} pcor(Cor, number \ TSSs\ | \{expression, genome \ context\} ) \end{aligned}$$

where Cor denotes the Pearson correlation of the model on test data and $\{expression, genome \ context\}$ subsumes all descriptors in these two descriptor categories.

In this approach, conditioning variables included only genomic descriptor of other categories. This process was repeated iteratively to obtain rank-based Spearman partial correlation values for each descriptor from each of the three categories, allowing us to assess their unique contributions to model performance.

Furthermore, model performance was quantified using correlation values, with missing entries assigned a value of zero. This adjustment ensured methodological consistency and preserved the genomic structure and model-specific feature distributions, particularly given that certain models exhibited limited performance for genes with specific characteristics.

## Assessing model generalizability across distinct cell types

### Selection of test and train samples

To assess model generalization, we defined a cell type-specific partitioning of the dataset into training and test samples based on cell type similarity. Instead of random partitioning, we selected test samples from cell types that were most distinct from the remaining ones. Distinctness was determined by computing Euclidean distances in principal component space based on the mean expression profiles of each cell type, calculated using the 5,000 most variable genes across all samples. Eight cell types with the largest distance to the centroid of the remaining cell types were selected as the test set (Additional file 1: Fig. S11a, b). The resulting partition comprises 36 test samples representing the most distinct cell types, while the remaining samples were used for training.

### Selection of gene subsets

To evaluate model performance under the cell type-specific partition, we selected 246 genes with distinct expression characteristics (table provided on Zenodo). Specifically, we identified the four cell types from the test set with the highest number of samples (Fig. 6b) in the new test–train split and computed the z-score of RNA counts per gene across cell types. More precisely, we first calculated the mean RNA counts per cell type and then the z-score across cell types. For each of the four cell types, the top 50 genes with the highest z-scores were selected as cell type-specific genes. In addition, we included 50 low-variant genes, defined as those with the lowest standard deviation across all cell types (minimum mean expected counts $\ge ~100$), representing genes with stable and ubiquitous expression. This resulted in a total of 246 unique genes due to partial overlap between cell type-specific sets.

### HAWAS-gene test

The HAWAS-gene approach leverages ML-based predictions of gene expression to identify genes with significant alterations between healthy and disease states. By comparing the predicted expression profiles of thousands of genes, this approach enables the discovery of potential disease-associated genes driven by epigenetic regulation (Algorithm 1).

**Figure:**
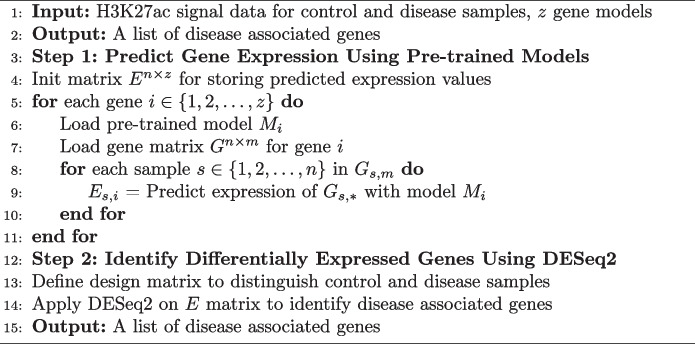
**Algorithm 1** HAWAS-gene test

In the first step, two best pre-trained ML models (CRE-RF and Binned-CNN) are used to predict gene expression counts based on H3K27ac signal data from both control and disease samples. For $z$ genes, there are $z$ corresponding models, where each model $M_i$ ($i \in \{1, 2, \dots , z\}$) is associated with an input matrix $G^{n \times m}$. Here, $n$ represents the total number of samples, including both control and disease groups, and $m$ represents the number of genomic features (regions) of the gene model $M_i$.

For each gene $i$, the model produces the predicted expression count and stores it in $E_{s,i}$. This process is repeated for all $z$ genes, and the final results are stored in the output matrix $E^{n\times z}$, where $n$ (rows) corresponds to the input samples, and $z$ (columns) represents the predicted expression values for all genes.

In the second step, differentially expressed genes between control and disease samples are identified using DESeq2 [65]. Specifically, a design matrix is constructed to model the two conditions: control and disease with sex included as a covariate. DESeq2 is then applied to the predicted expression count matrix $E^{n\times z}$. The test returns a list of disease-associated genes based on statistically significant expression changes.

### HAWAS-region test

While identifying HAWAS genes provides critical insights, understanding which regulatory elements contribute to these expression changes is essential for deciphering disease mechanisms. The HAWAS-region test extends the analysis to the level of gene regulatory regions by quantifying the contribution of individual regions (features) to gene expression by using ISP. This method identifies regulatory elements whose influence on gene expression differs between healthy and disease conditions (Algorithm 2).

**Figure:**
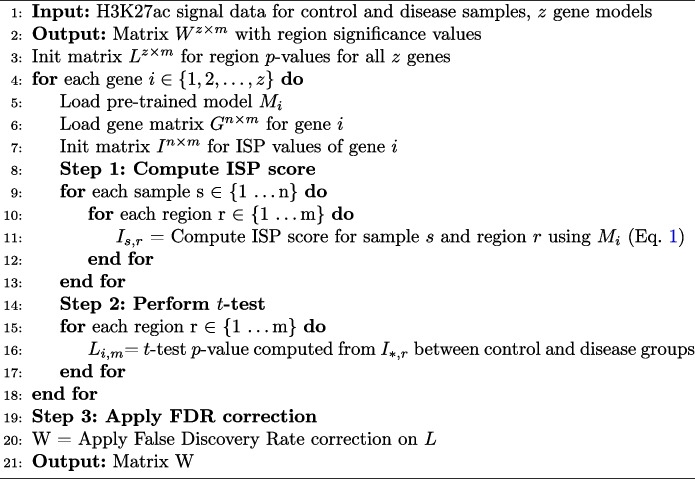
**Algorithm 2** HAWAS-region test

In detail, the input of this method are $z$ gene models. The input for each gene *i* is a $G^{n\times m}$ matrix with H3K27ac signal with $n$ rows representing samples and $m$ columns representing regions. In the first step, the ISP score is calculated for each sample $s \in \{1, 2, \dots , n\}$ for all regions $r \in \{1, 2, \dots , m\}$ of gene *i* using the ISP formula (Eq. 1) resulting in a matrix $I^{s\times r}$. Importantly, these per individual ISP values represent the model’s estimated regulatory contribution of each CRE to gene expression rather than the raw H3K27ac signal itself. For notational simplicity we assume here that each gene *i* has the same number of regions *m*. In the second step, a *t*-test is performed between the control and disease groups for gene *i* across all $m$ regulatory regions and the resulting *p*-values are stored in $L^{i\times m}$. *t*-test is chosen because it is well-suited for comparing continuous values, e.g., region importance scores. A two-sided test is used. Hence, after the *t*-test there are *m* many *p*-values for the gene *i*. This process is repeated for all $z$ genes and there exist $z \times m$ many *p*-values that are stored in list $L$. In the third step, a false discovery rate (FDR) correction is applied on the *p*-values from list $L$ to adjust for multiple testing [80]. Hence, the output is list *W* which contains significant regions with their adjusted *p*-values.

### Transcription factor enrichment and survival analysis

To investigate the potential regulatory mechanisms underlying disease-associated regions, TF motif enrichment analysis was performed using the PASTAA algorithm [46]. This analysis was done by ranking the significant regions ascendingly by their p-value and calculating the TF affinities with TRAP [81] based on a motif collection of 818 position frequency matrices from JASPAR 2022 [82], HOCOMOCO v11 [83] and the work of Kheradpour and Kellis [84], available under https://github.com/SchulzLab/STARE/blob/main/PWMs/2.2/Jaspar_Hocomoco_Kellis_human_transfac.txt. TFs with an FDR from PASTAA below 0.01 were considered significant. This procedure was applied to the significant regulatory regions identified by the HAWAS-region test using the CRE-RF model. Enrichment results were filtered based on a false discovery rate (FDR) threshold of 0.01, and top-ranking TFs were further examined based on literature evidence for relevance to leukemia and colon cancer.

For survival analysis, the Survival Genie2 tool [47] was used, and lymphoid leukemia was selected as the disease of interest. We chose the TARGET-ALL-P2-Bone-Marrow dataset, with overall survival as outcome type, took primary tumor types and considered all available samples. The top 10 leukemia TFs identified from the motif enrichment analysis were evaluated across these datasets. Patients were stratified based on the median expression levels of each TF, and survival differences between groups were assessed using the log-rank test. TFs with *p*-values $\le 0.05$ were considered significant.

### HAWAS on CLL and colon cancer data

A subset of available healthy and disease samples was selected, and samples identified as outliers by PCA were removed. There were some modification in the pipeline based on the data characteristics. First, for each of the 27,385 shared genes, CRE-RF and Binned-CNN models were used to predict expression values in the selected samples. Second, before performing the DESeq2 analysis, genes with fewer than 10 counts across all samples were removed. Third, since the size factor-normalized read counts are used in this study the size factor variable in the DESeq2 pipeline was set to 1 to prevent normalization. Fourth, sex was included as a covariate in the analysis. The metadata predicted by EpiClass [85] was used for the covariate, more precisely the column “EpiClass_pred_Sex”.

To determine related regions (features) of the significant genes of the HAWAS-gene test, the HAWAS-region test was used with an adjusted *p*-value cutoff of 0.05. In cases where redundant regions remained after FDR correction, the one with the smallest adjusted *p*-value was selected. The identified HAWAS regions were further analyzed to uncover potential TFs involved in disease-specific gene regulation using PASTAA [46]. This analysis identified 30 significant TFs for CLL and 2 significant TFs for colon cancer. Moreover, to assess the biological relevance of the HAWAS genes, comparisons were made with known CLL genes from the DisGeNET database [45] (Fig. 7d) and known colon cancer genes (Additional file 1: Fig. S14c).

### CLL-genes via nearest gene and gABC

To have a comparison of the HAWAS-gene test to conventional approaches, regions with differential H3K27ac signal between control and CLL were called with DiffBind (v.3.4.11) [49]. As peaks, the ENCODE CREs were taken and the ChIP-seq input was given as background for each sample. Peaks with FDR $\le 0.05$ and an absolute log fold-change of $\ge$ 0.585 were considered differential. To then link the differential peaks to genes, two approaches were used. First, each differential peak was associated to its nearest gene within 100 kb based on the distance to the 5’ TSS with the closest function from bedtools [76]. Second, differential peaks were mapped to all genes they form interactions with according to the gABC-score. To get the gABC-score, STARE (v.1.0.4) [50] was run for each condition with all ENCODE CREs as candidate enhancers and their average fold-change H3K27ac ChIP-seq signal as enhancer activity. As contact data, an average Hi-C matrix was used [36]. The window size was set to 1 MB, the score cutoff to 0.02 and regions known to accumulate anomalous signal were removed [86, 87]. If a gene was predicted to form an interaction with a differential peak in either condition, it was added to the gABC-score gene set. For both approaches, only genes that were also considered for model learning were kept.

## Supplementary Information

Additional file 1. Supplementary Table and figures including benchmarking, validation and HAWAS analyses.

## Data Availability

The trained models of the two best-performing approaches (CRE-RF and Binned-CNN), as well as all necessary data and files required to execute the source code and the pipeline, are available on Zenodo [88]. Training data was taken from the IHEC EpiATLAS portal (https://ihec-epigenomes.org/epiatlas/data/). All other data was taken from public resources as cited and indicated in the methods. The code used for the analyses is publicly available on GitHub [89], and the statistic version of it is archived on Zenodo [90] at https://doi.org/10.5281/zenodo.20270475 under an MIT license.
