# Supplementary material for: Comparing machine learning methods predicting transcriptome from epigenome with applications to association studies
Source: Genome Biol. 2026 Jul 13;27:222. doi: 10.1186/s13059-026-04131-w (PMC13361598; doi:10.1186/s13059-026-04131-w)
Supplement: Supplementary file 1 — Additional file 1. Supplementary Table and figures including benchmarking, validation and HAWAS analyses. [file 13059_2026_4131_MOESM1_ESM.pdf]

Additional File 1: Supplementary figures and tables

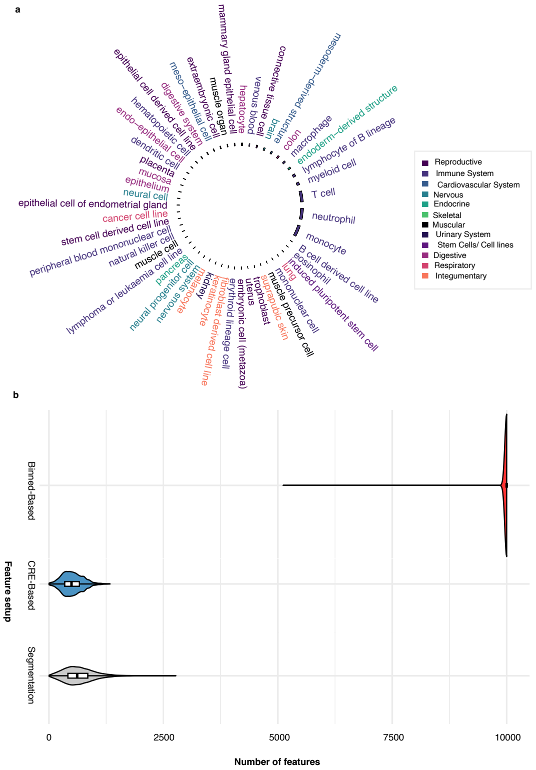

Fig. S1 Number of samples per cell type and distribution of input features for different methods. **(a)** Circos plot showing the number of samples of each cell type, colored by the higher-level lineage. The plot was generated with the circlize R package [1]. **(b)** Violin plots show distribution of input features for different feature setups.

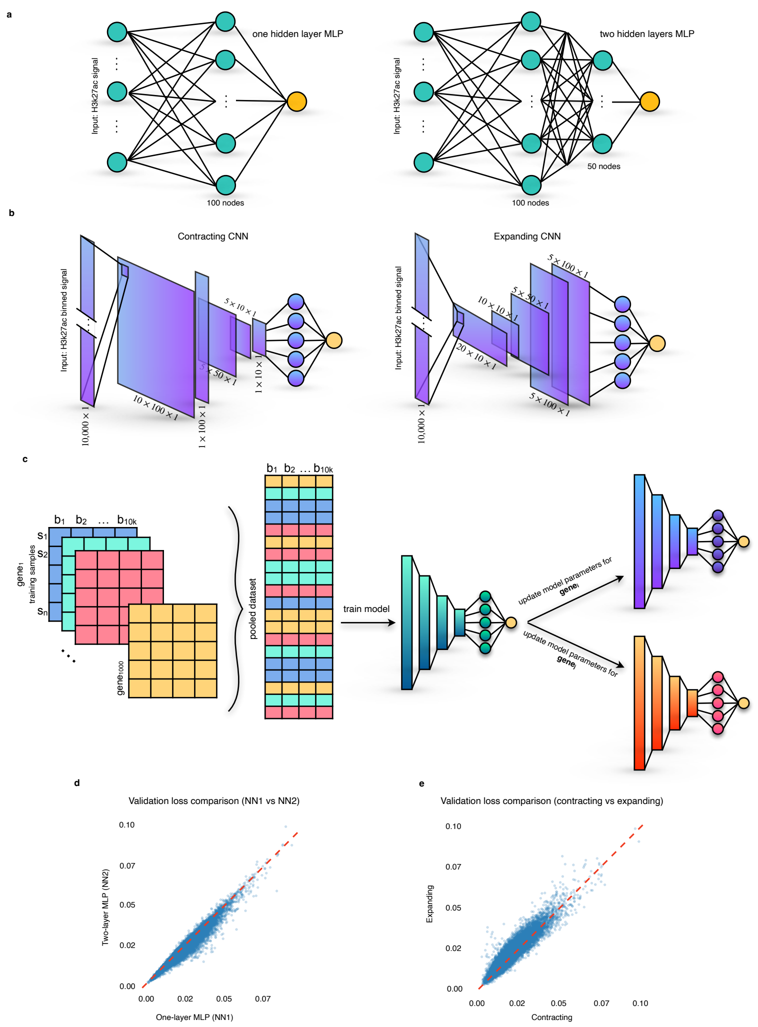

Fig. S2 Architectures of the Multi-Layer Perceptron (MLP) and convolutional Neural Network (CNN) models and warm start strategy for CNN (a) One the left side the MLP model with a single hidden layer consists of 100 nodes, fully connected to both the input layer and the output neuron. This model processes the H3K27ac signal as input and applies ReLU activation functions in the hidden layer, while the output layer utilizes a linear activation function for continuous predictions. The output of the model is the predicted RNA-seq expression count. On the right site the MLP model with two hidden layers features 100 nodes in the first hidden layer and 50 nodes in the second hidden layer, and other features are similar to one-layer MLP. **(b)** On the right site, architecture of the contracting convolutional neural network (CNN), where the kernel sizes progressively decrease from the input layer to the output layer. On the left site, expanding CNN architecture, characterized by progressively larger kernel sizes from the input to the output layer. Both topologies include a fully connected dense layer with 5 hidden nodes linking the convolutional block to the output regression node which is designed to predict RNA expression counts. The ReLU activation function is applied to all layers except the first and last, where a sigmoidal activation function is used instead. Layer sizes are indicated in the figure. **(c)** A heuristic warm start approach was used for weight initialization of CNN models. Three datasets were created, each containing 1,000 randomly selected genes along with their corresponding biological samples. The contracting and expanding kernel CNNs were independently trained on these datasets, yielding optimized models for each subset.**(d)** Validation loss comparison between one-layer MLP and two-layer MLP. **(e)** Validation loss comparison between contracting and expanding CNN.

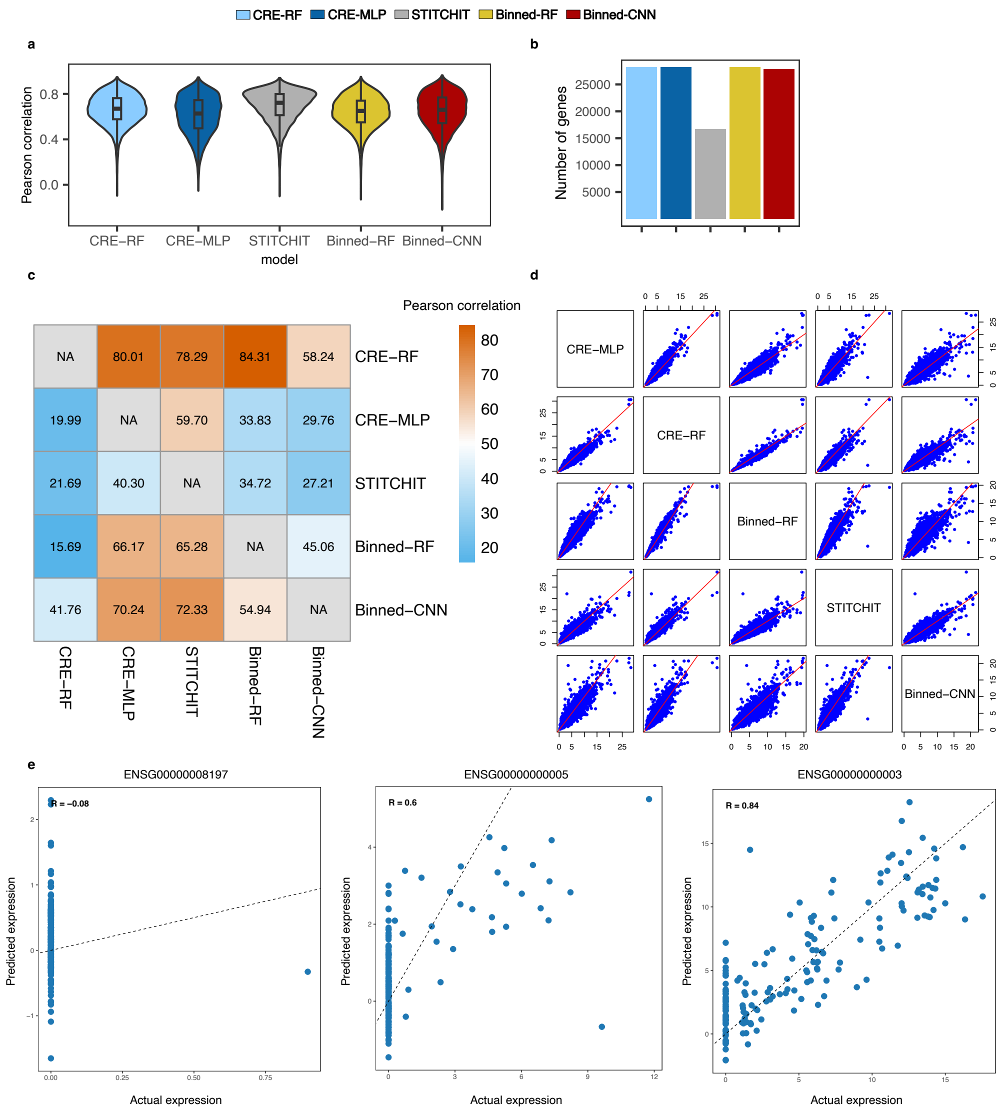

Fig. S3 Performance assessment of the five machine learning approaches (a) Violin plot of the Pearson correlation for all genes including *high* (≥ 0.7), *intermediate* (≥ 0.3) and *fail* (< 0.3) Center lines of the included box plots are the median, boxlimits indicate the interquartile range (IQR), whiskers 1.5x IQR, outliers are removed. **(b)** Barplot of the number of the total learned models **(c)** Heatmap for the pairwise Pearson correlation comparison of the approaches, showing the percentage for which the method in the row outperforms the method in the column **(d)** MSE scatterplots for pairwise method comparison. Correlation and MSE are always estimated on the test set. **(e)** Comparison of predicted (STITCHIT) and measured gene expression for example genes representing high, intermediate, and fail performance classes.

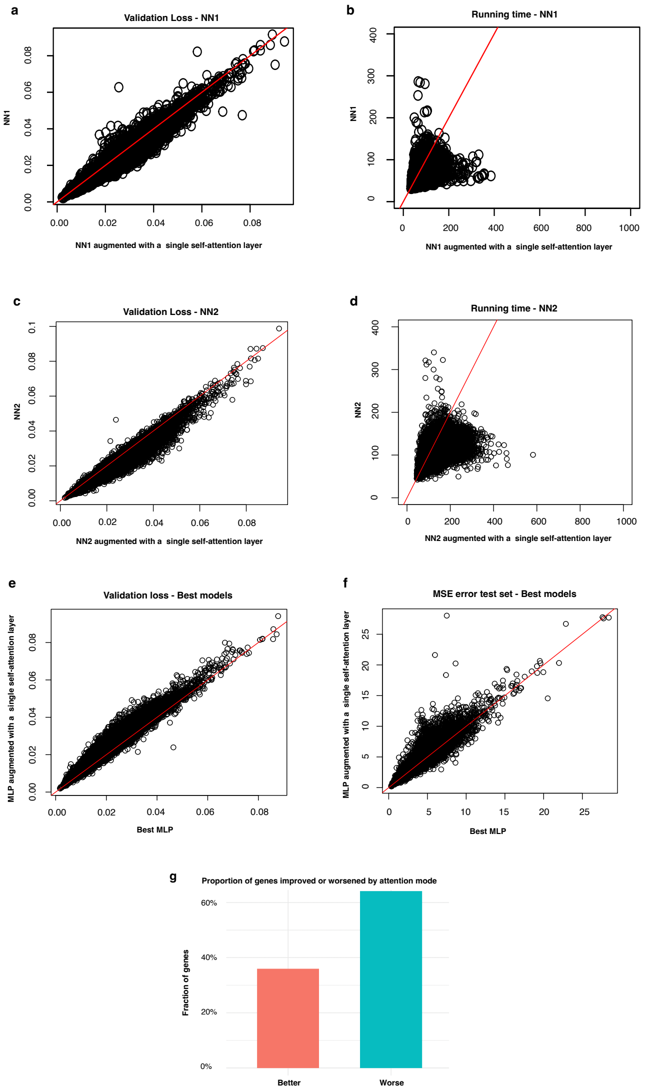

Fig. S4 Comparison between baseline MLPs and MLPs augmented with a single self attention layer. (**a,b**) Comparison between NN1 and NN1 with self attention in terms of validation loss (a) and running time (b). Adding a self-attention layer slightly increased computational time but did not notably improve validation loss. (**c,d**) Comparison between NN2 and NN2 with self attention for validation loss (c) and running time (d), showing a similar pattern for the running time but more genes have a higher validation loss in the NN2 with self attention. After these individual comparisons, the best performing MLP was selected from NN1 and NN2, and the best attention augmented MLP was selected from NN1 with attention and NN2-with-attention. (**e,f**) Comparison between these two best models baseline MLP vs. attention augmented MLP based on validation loss (e) and mean squared error (MSE). **(f)** The attention augmented model exhibited marginally higher validation loss and MSE, indicating no consistent advantage from adding attention. (**g**) Fraction of genes whose prediction performance improved or worsened after adding the attention layer, showing that only a 40% benefited while 60% slightly degraded. Quantitatively, the attention-augmented models achieved a median test correlation of 0.627, median test MSE of 4.352, and median validation loss of 0.0233, whereas the corresponding baseline MLPs achieved 0.627, 4.194, and 0.02, respectively.

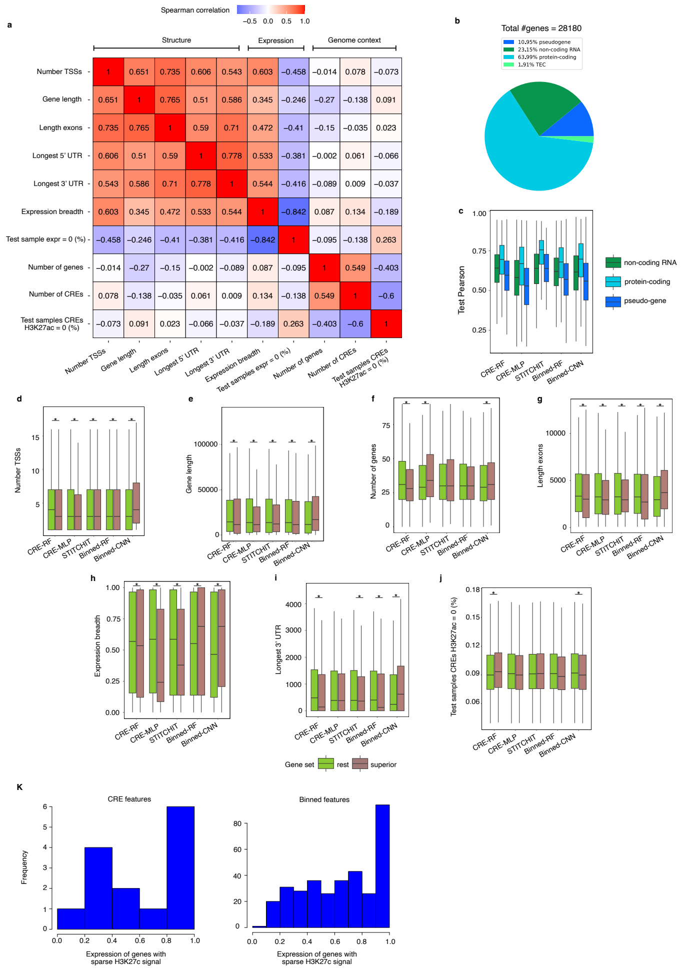

Fig. S5 Investigation of gene characteristics that affect model performance. (a) Spearman correlation heatmap for the gene descriptors. Descriptors that cluster together according to their correlation are grouped into three categories *structure*, *expression* and *genome context*. **(b)** Pie chart for the biotypes of the genes that have $\geq$ 2 expression variance and 90% non-zero values across samples in the RNA data. **(c)** Boxplot for the model performances (Pearson correlation) for each method on the subsets of genes that are non-coding, protein-coding and pseudogenes. **(d-i)** Boxplot for a gene descriptor showing the genes for which a method has the lowest MSE compared to the other methods (superior) and all the remaining genes (rest). The descriptors are **(d)** number of TSSs, **(e)** gene length, **(f)** number of other genes in the 1 MB gene window, **(g)** total exon length, **(h)** expression breadth, i.e. fraction of cell types/tissues (n = 58) in the entire dataset where the target gene is expressed (TPM $\geq$ 0.5), **(i)** length of the longest 3' UTR. For boxplots **(c-i)** the center line indicates the median, boxlimits correspond to IQR, whiskers to 1.5x IQR. An unpaired Mann-Whitney U Test was performed between the "superior" and "rest" gene sets for each gene descriptor and each method separately. A p-value $\leq$ 0.05 is indicated with an asterisk.Colours in **(b)** and **(c)** taken from the colorcet package based on Glasbey et al.. Pearson and MSE values were estimated on the test set. **(k)** Histograms showing the expression of genes with high sparsity in H3K27ac signal for CRE (left panel) and Binned features. Data for STITCHIT are not shown, as only three genes met the sparsity criterion. For all the feature setups, a threshold of 80% was applied to the fraction of H3K27ac signal to identify highly sparse genes.

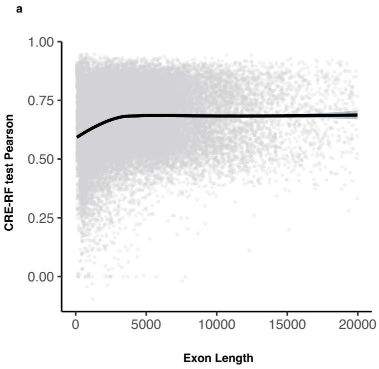

Fig. S6 Comparing CRE-RF test correlation with different descriptors. (a) Scatter plot comparing the exon length and model performance of the CRE-RF (Pearson correlation), here 300 outlier genes with exon length greater than 20,000 were excluded. **(b)** Scatter plot comparing the length of longest 5' UTR and model performance of the CRE-RF (Pearson correlation), here 1,205 outlier genes with longest 5' UTR greater than 700 were excluded.

Table 1 List of transcription factors (TFs) identified by motif enrichment analysis in CRE-RF regions. The table lists 20 remaining TFs associated with CRE-RF regions (FDR < 0.01). The top 10 TFs are shown in Fig. 6e. The column "Leukemia Ref." indicates whether each TF has previously been reported in leukemia-related studies, with corresponding references[17–30].

TF	Enrichment FDR	Leukemia Ref.
GSC2	0.006	Giacomo *et al.* (2015)
ONECUT1	0.007	–
KLF5	0.008	Diakiw *et al.* (2012)
IRF8	0.008	Slager *et al.* (2013)
VEZF1	0.008	–
FLI1::FOXI1	0.008	–
RAX	0.008	Ruvolo *et al.* (2000)
RELA	0.009	Mulligan *et al.* (2023)
ZNF322	0.009	–
ZNF417	0.009	Martins *et al.* (2024)
VAX1	0.009	Burkhardt *et al.* (2013)
HSF4	0.009	Lyu *et al.* (2022)
MSC	0.009	Fallati *et al.* (2022)
TCF3	0.009	Zerkalenkova *et al.* (2023)
ZNF502	0.009	Zhang *et al.* (2023)
RHOXF1	0.009	–
MEF2B	0.009	El Jamal *et al.* (2019)
MTF1	0.009	Li *et al.* (2023)
MLXIPL	0.009	–
VSX2	0.009	Heller *et al.* (2016)

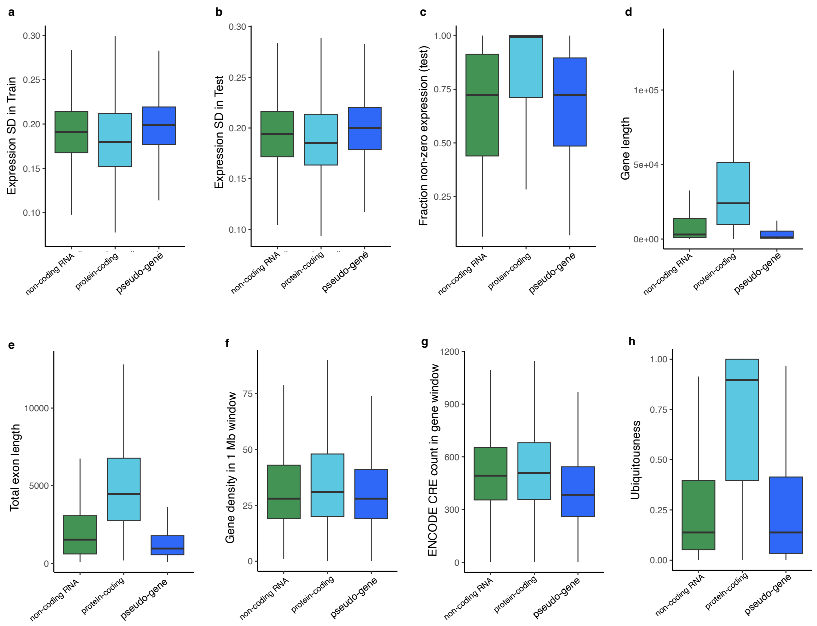

Fig. S7 Comparison of gene types across gene-level descriptors. (a-h) Comparison of expression and gene descriptors for protein-coding, non-coding RNA, and pseudogene biotypes. Features include expression variance in train and test, gene and exon length, gene density, number of CREs, and ubiquitousness. Protein-coding genes display characteristic shifts in multiple descriptors, potentially explaining their consistently higher predictive performance. SD: standard deviation.

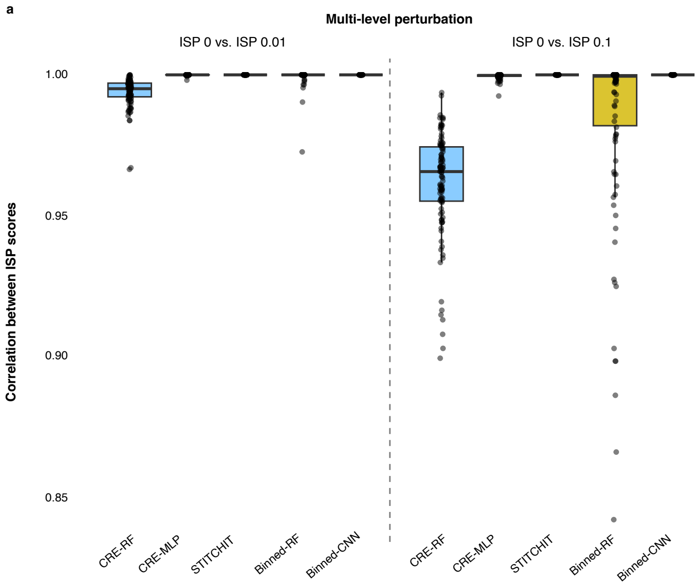

Fig. S8 Multi-level ISP comparison. (a) To evaluate the stability of the in silico perturbation (ISP) score with respect to the perturbation magnitude, we repeated the perturbation analysis using smaller non-zero scaling factors (0.01 and 0.1) instead of setting the signal of a region to zero. The left panel shows the correlation between ISP scores obtained from perturbations set to 0 and 0.01, while the right panel shows the correlation between ISP scores from perturbations set to 0 and 0.1, across all five models (CRE-RF, CRE-MLP, STITCHIT, Binned-RF, Binned-CNN). The consistently high correlations ($r > 0.95$) observed for all models demonstrate that the ISP measure is highly stable and robust to the choice of perturbation level.

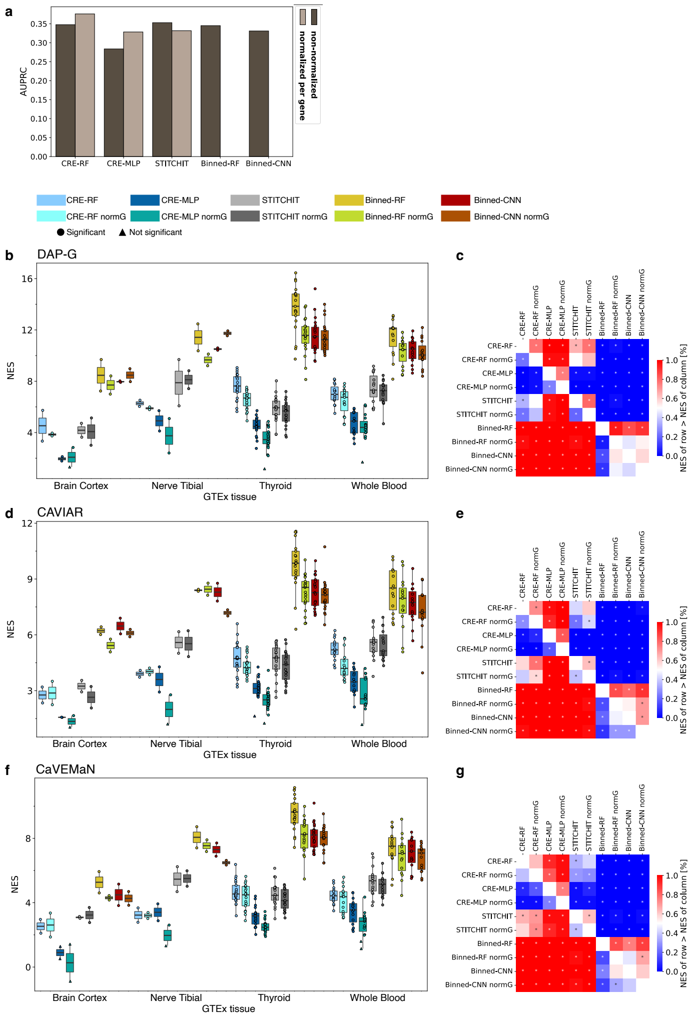

Fig. S9 Model comparison using CRISPRi and eQTL data. (a) Barplot with the area under the precision recall curve (AUPRC) of all models based on validated enhancer-gene interactions [3]. An *in silico* perturbation approach was implemented to assess the importance of an enhancer-gene interaction for a model. Two score calculations were tested: ISP and ISP_{normG}. The AUPRC was calculated based on the set of interactions for which all models could produce a score for, namely 1,099 tested interactions out of which 198 were significant. For Binned-RF and Binned-CNN ISP_{normG} was tested but is not shown, due to their feature space. Using the fused regions for normalization further reduced the set of interactions all model could produce a score for drastically, and normalizing across all bins was not computationally feasible. **(b)** Normalized enrichment score (NES) of the models for enhancer-gene interactions that are supported by eQTL-gene pairs from GTEx [4], that were fine-mapped with DAP-G [5]. NES and p-value calculated with GSEApy [6–8]. The boxplots are formed by the NES of the EpiATLAS samples that were matched to the respective GTEx tissue (boxplot center line is the median, boxlimits show the inter-quartile range, whiskers up to 1.5x interquartile range; significant means enrichment p-value $\leq$ 0.05). For all models both score calculations were tested. Here, ISP_{normG} was possible for binned-RF and binned-CNN with the fused regions per gene, since the top 100,000 scored interactions were taken per model and not jointly. **(c)** Pairwise model comparison of the NES from (b) across all EpiATLAS samples. An asterisk indicates a significant difference, assessed with a two-sided Wilcoxon signed-rank test. **(d,e)** equivalent to (b) and (c) but for eQTLs fine-mapped with CAVIAR [9]. For **(f,g)** the eQTLs fine-mapped with CaVEMaN [10] were used.

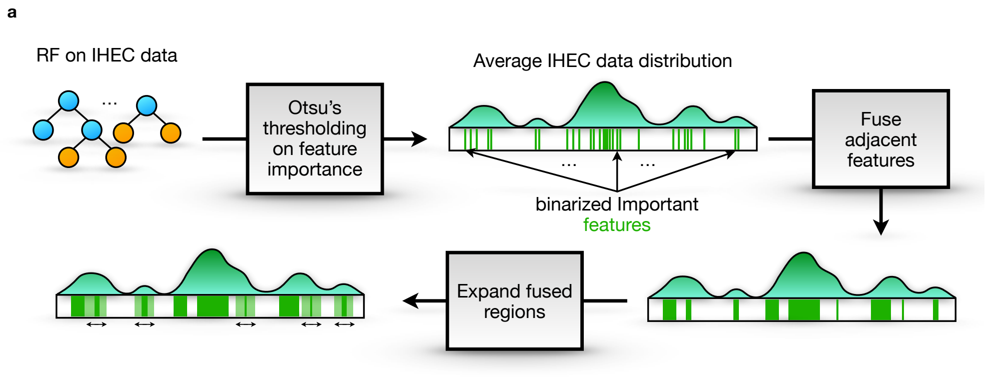

Fig. S10 Schematic illustration of feature fusion in Binned-CNN models. **(a)** Binned-RF are recruited to determine global feature importance for each gene. Then, Otsu's thresholding method is deployed to binarize feature importance values, classifying genomic bins as 'active' (1) or 'inactive' (0). To ensure meaningful feature selection, adjacent active bins are fused if they are separated by no more than five inactive bins, forming larger contiguous regions. Finally, these fused regions are further expanded to a minimum length of 1kb, through merging nearby (fused) regions, to form regions used for ISP analysis of the model.

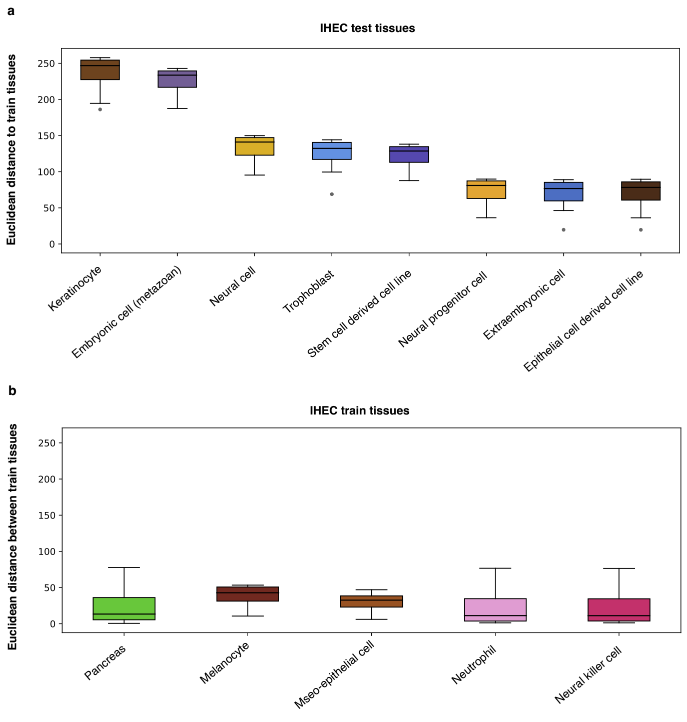

Fig. S11 Choosing test cell types and showing number of samples per test cell type. (a) Boxplots show the Euclidean distances between samples of each test cell type and all training cell types. **(b)** Distance distributions for five randomly selected training cell types, showing Euclidean distances between samples within the training set.

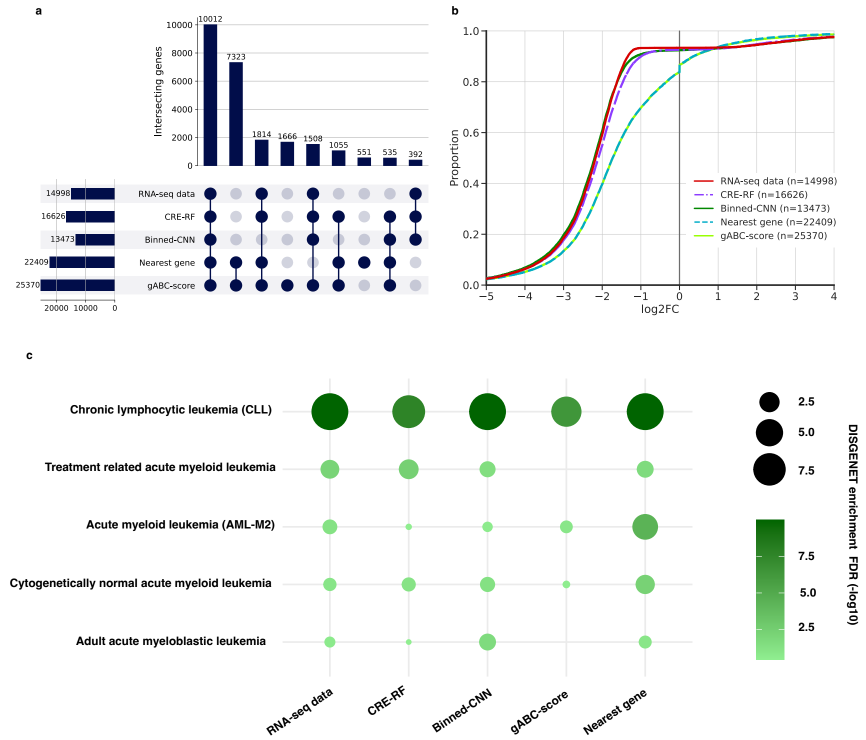

Fig. S12 Comparison of genes found by the HAWAS-gene test and genes found by conventional approaches. RNA-seq data: DEGs identified by DESeq2 [11] from the RNA-seq data. CRE-RF and Binned-CNN: Genes found with the HAWAS-gene test based on the CRE-RF and Binned-CNN models, respectively. Nearest gene: Genes that were closest to differentially acetylated regions identified with DiffBind [12], with a maximum distance of 100 kb. gABC-score: Genes that were associated to differentially acetylated regions according to the interactions predicted with the gABC-score [13]. All sets were limited to the 28,180 genes that were considered for model training. **(a)** UpSet plot showing the overlap of the gene sets, limited to the nine largest intersections [14]. **(b)** Cumulative distribution of the log2FC of the gene sets, as measured by the RNA-seq data. A positive log2FC indicates downregulation in CLL and vice versa. **(c)** Dot plot illustrating the enrichment of known leukemia genes (DisGeNET database [15]) among the gene sets (enrichment test -$\log_{10}$ FDR).

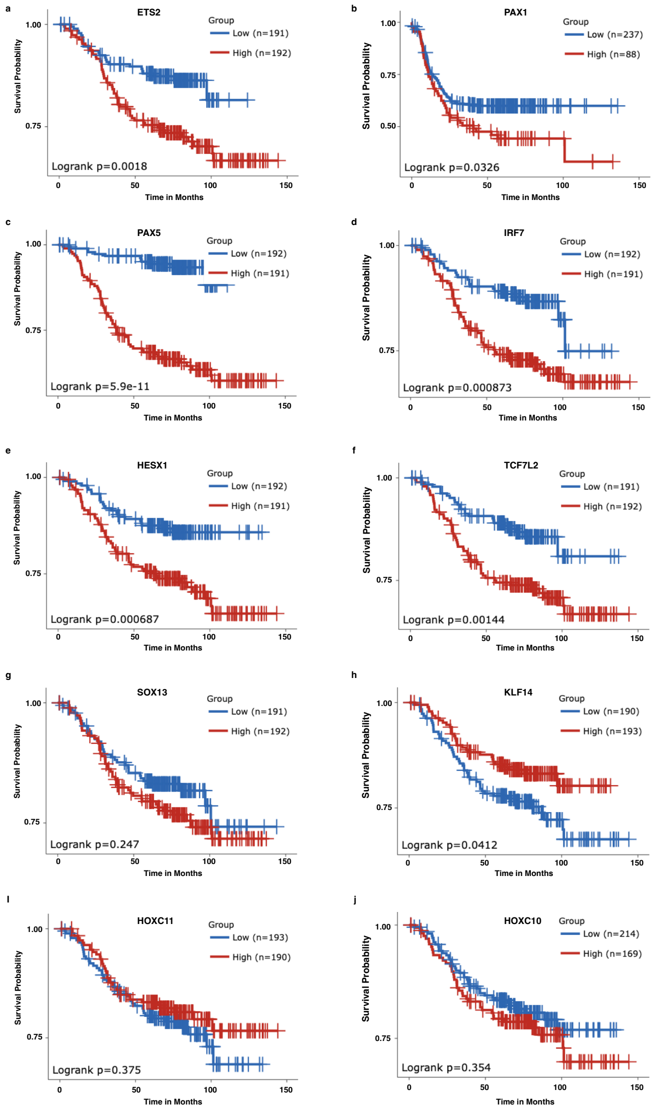

Fig. S13 Kaplan-Meier survival analysis plots (a–j) for the top 10 TFs between control and CLL samples done with SurvivalGenie2.0 while using the TARGET-ALL-P2-Bone-Marrow dataset [16]**.** Each panel corresponds to a specific TF: **(a)** ETS2, **(b)** PAX1, **(c)** PAX5, **(d)** IRF7, **(e)** HESX1, **(f)** TCF7L2, **(g)** SOX13, **(h)** KLF14, **(i)** HOXC11, **(j)** HOXC10.

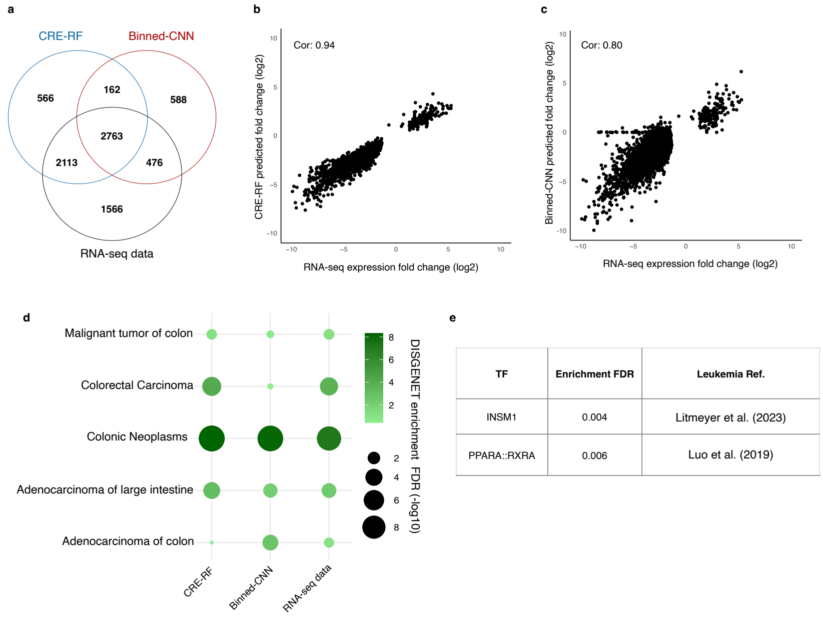

TF	Enrichment FDR	Leukemia Ref.
INSM1	0.004	Litmeyer et al. (2023)
PPARA::RXRA	0.006	Luo et al. (2019)

Fig. S14 Model-based HAWAS-gene analysis on Grade 1 Colon Adenocarcinoma samples. (a) Venn diagram showing the overlap of significant genes identified from RNA-seq, CRE-RF, and Binned-CNN predictions (6,918, 5,604, and 3,989 significant genes, respectively). **(b, c)** Scatter plots comparing $\log_2$ fold changes between measured RNA-seq expression and model-predicted expression for CRE-RF and Binned-CNN, respectively (R = 0.94 for CRE-RF and R = 0.80 for Binned-CNN). **(d)** Dot plot illustrating the overlap of known colon genes (DisGeNET database [15]) and colon genes detected by CRE-RF, Binned-CNN, and RNA-seq expression analysis (enrichment test -$\log_{10}$ FDR). **(e)** Table showing the 2 enriched transcription factors (TFs) identified by motif enrichment with PASTAA [31] on 583 CREs associated with colon using CRE-RF models. PASTAA enrichment FDR and supporting references are shown in the second and last columns [32, 33].

References

[1] Gu Z, Gu L, Eils R, Schlesner M, Brors B. circlize implements and enhances circular visualization in R. Bioinformatics. 2014 06;30(19):2811–2812. https://doi.org/10.1093/bioinformatics/btu393. https://academic.oup.com/bioinformatics/article-pdf/30/19/2811/49872421/bioinformatics_30_19_2811.pdf.

[2] Glasbey C, Van Der Heijden G, Toh VFK, Gray A. Colour displays for categorical images. Color Research & Application. 2007 Aug;32(4):304–309. https://doi.org/10.1002/col.20327.

[3] Gschwind AR, Mualim KS, Karbalayghareh A, Sheth MU, Dey KK, Jagoda E, et al. An encyclopedia of enhancer-gene regulatory interactions in the human genome [preprint]. bioRxiv. 2023 Nov;https://doi.org/10.1101/2023.11.09.563812.

[4] Consortium TG, Aguet F, Anand S, Ardlie KG, Gabriel S, Getz GA, et al. The GTEx Consortium atlas of genetic regulatory effects across human tissues. Science. 2020;369(6509):1318–1330. https://doi.org/10.1126/science.aaz1776. https://www.science.org/doi/pdf/10.1126/science.aaz1776.

[5] Wen X, Lee Y, Luca F, Pique-Regi R. Efficient Integrative Multi-SNP Association Analysis via Deterministic Approximation of Posteriors. The American Journal of Human Genetics. 2016;98(6):1114–1129. https://doi.org/10.1016/j.ajhg.2016.03.029.

[6] Mootha VK, Lindgren CM, Eriksson KF, Subramanian A, Sihag S, Lehar J, et al. PGC-1α-responsive genes involved in oxidative phosphorylation are coordinately downregulated in human diabetes. Nature Genetics. 2003 Jul;34(3):267–273. https://doi.org/10.1038/ng1180.

[7] Subramanian A, Tamayo P, Mootha VK, Mukherjee S, Ebert BL, Gillette MA, et al. Gene set enrichment analysis: A knowledge-based approach for interpreting genome-wide expression profiles. Proceedings of the National Academy of Sciences. 2005 Oct;102(43):15545–15550. https://doi.org/10.1073/pnas.0506580102.

[8] Fang Z, Liu X, Peltz G. GSEApy: a comprehensive package for performing gene set enrichment analysis in Python. Bioinformatics. 2023 Jan;39(1):btac757. https://doi.org/10.1093/bioinformatics/btac757.

[9] Hormozdiari F, Kostem E, Kang EY, Pasaniuc B, Eskin E. Identifying Causal Variants at Loci with Multiple Signals of Association. Genetics. 2014 08;198(2):497–508. https://doi.org/10.1534/genetics.114.167908. https://academic.oup.com/genetics/article-pdf/198/2/497/42132777/genetics0497.pdf.

[10] Brown AA, Viñuela A, Delaneau O, Spector TD, Small KS, Dermitzakis ET. Predicting causal variants affecting expression by using whole-genome sequencing and RNA-seq from multiple human tissues. Nature Genetics. 2017;49(12):1747–1751. https://doi.org/10.1038/ng.3979.

[11] Love MI, Huber W, Anders S. Moderated estimation of fold change and dispersion for RNA-seq data with DESeq2. Genome Biology. 2014 Dec;15(12):550. https://doi.org/10.1186/s13059-014-0550-8.

[12] Ross-Innes CS, Stark R, Teschendorff AE, Holmes KA, Ali HR, Dunning MJ, et al. Differential oestrogen receptor binding is associated with clinical outcome in breast cancer. Nature. 2012 Jan;481(7381):389–393. https://doi.org/10.1038/nature10730.

[13] Hecker D, Behjati Ardakani F, Karollus A, Gagneur J, Schulz MH. The adapted Activity-By-Contact model for enhancer-gene assignment and its application to single-cell data. Bioinformatics (Oxford, England). 2023 Jan;p. btad062. https://doi.org/10.1093/bioinformatics/btad062.

[14] Lex A, Gehlenborg N, Strobelt H, Vuillemot R, Pfister H. UpSet: Visualization of Intersecting Sets. IEEE Transactions on Visualization and Computer Graphics. 2014 Dec;20(12):1983–1992. https://doi.org/10.1109/TVCG.2014.2346248.

[15] Piñero J, Ramírez-Anguita JM, Saüch-Pitarch J, Ronzano F, Centeno E, Sanz F, et al. The DisGeNET knowledge platform for disease genomics: 2019 update. Nucleic Acids Research. 2019 11;48(D1):D845–D855. https://doi.org/10.1093/nar/gkz1021. https://academic.oup.com/nar/article-pdf/48/D1/D845/31697865/gkz1021.pdf.

[16] Dwivedi B, Mumme H, Satpathy S, et al. Survival Genie, a web platform for survival analysis across pediatric and adult cancers. Scientific Reports. 2022;12:3069. https://doi.org/10.1038/s41598-022-06841-0.

[17] Di Giacomo D, La Starza R, Barba G, Pierini V, Baldazzi C, Storlazzi CT, et al. 4q12 translocations with GSX2 expression identify a CD7(+) acute myeloid leukaemia subset. British Journal of Haematology. 2015 October;171(1):141–145. Epub 2015 Mar 30. https://doi.org/10.1111/bjh.13368.

[18] Diakiw SM, Kok CH, To LB, Lewis ID, Brown AL, D'Andrea RJ. The granulocyte-associated transcription factor Krüppel-like factor 5 is silenced by hypermethylation in acute myeloid leukemia. Leukemia Research. 2012 January;36(1):110–116. Epub 2011 Oct 11. https://doi.org/10.1016/j.leukres.2011.09.013.

[19] Slager SL, Achenbach SJ, Asmann YW, Camp NJ, Rabe KG, Goldin LR, et al. Mapping of the IRF8 gene identifies a 3'UTR variant associated with risk of chronic lymphocytic leukemia but not other common non-Hodgkin

lymphoma subtypes. Cancer Epidemiology, Biomarkers & Prevention. 2013 March;22(3):461–466. Epub 2013 Jan 10. https://doi.org/10.1158/1055-9965.EPI-12-1217.

[20] Ruvolo P, Gao F, Deng X, May WS. Rax, the cellular pkr activator, potentiates ceramide-induced apoptosis in hematopoietic cells. Experimental Hematology. 2000;28(7, Supplement 1):107. https://doi.org/10.1016/S0301-472X(00)00420-3.

[21] Mulligan EA, Ennis C, McCann K, Quinn S, Grigoriadis A, Das S, et al. Expression and Activity of the NF-κB Subunits in Chronic Lymphocytic Leukaemia: A Role for RelB and Non-Canonical Signalling. Cancers. 2023 September;15(19):4736. Epub 2023 Sep 26. https://doi.org/10.3390/cancers15194736.

[22] Martins F, Rosspopoff O, Carlevaro-Fita J, Forey R, Offner S, Planet E, et al. A Cluster of Evolutionarily Recent KRAB Zinc Finger Proteins Protects Cancer Cells from Replicative Stress-Induced Inflammation. Cancer Research. 2024 March;84(6):808–826. https://doi.org/10.1158/0008-5472.CAN-23-1237.

[23] Burkhardt UE, Hainz U, Stevenson K, Goldstein NR, Pasek M, Naito M, et al. Autologous CLL cell vaccination early after transplant induces leukemia-specific T cells. Journal of Clinical Investigation. 2013 September;123(9):3756–3765. Epub 2013 Aug 5. https://doi.org/10.1172/JCI69098.

[24] Lyu C, Wang Q, Yin X, Li Z, Wang T, Wang Y, et al. Clinical significance and potential mechanism of heat shock factor 1 in acute myeloid leukemia. Aging (Albany NY). 2022 September;14(17):7026–7037. Epub 2022 Sep 6. https://doi.org/10.18632/aging.204267.

[25] Fallati A, Di Marzo N, D'Amico G, Dander E. Mesenchymal Stromal Cells (MSCs): An Ally of B-Cell Acute Lymphoblastic Leukemia (B-ALL) Cells in Disease Maintenance and Progression within the Bone Marrow Hematopoietic Niche. Cancers (Basel). 2022 July;14(14):3303. https://doi.org/10.3390/cancers14143303.

[26] Zerkalenkova E, Menchits Y, Borkovskaia A, Sokolova S, Soldatkina O, Mikhailova E, et al. TCF3 gene rearrangements in pediatric B-cell acute lymphoblastic leukemia–A single center experience. International Journal of Laboratory Hematology. 2023 August;45(4):533–540. Epub 2023 Apr 14. https://doi.org/10.1111/ijlh.14072.

[27] Zhang X, Chen X, Sun D, Song N, Li M, Zheng W, et al. ENAH-202 promotes cancer progression in oral squamous cell carcinoma by regulating ZNF502/VIM axis. Cancer Medicine. 2023 November;12(22):20892–20905. Epub 2023 Oct 30. https://doi.org/10.1002/cam4.6652.

[28] El Jamal SM, Grada Z, El Dinali MH, Zhou H, Hassan SY, Saad AG, et al. MEF2B is a member of the BCL6 gene transcriptional complex and induces its

expression in diffuse large B-cell lymphoma of the germinal center B-cell-like type. Laboratory Investigation. 2019 April;99(4):539–550. Epub 2018 Nov 16. https://doi.org/10.1038/s41374-018-0152-2.

[29] Li Y, Kan X. Cuproptosis-Related Genes MTF1 and LIPT1 as Novel Prognostic Biomarker in Acute Myeloid Leukemia. Biochemical Genetics. 2024 April;62(2):1136–1159. Epub 2023 Aug 10. https://doi.org/10.1007/s10528-023-10473-y.

[30] Heller G, Topakian T, Altenberger C, Cerny-Reiterer S, Herndlhofer S, Ziegler B, et al. Next-generation sequencing identifies major DNA methylation changes during progression of Ph^+ chronic myeloid leukemia. Leukemia. 2016 September;30(9):1861–1868. Epub 2016 May 23. https://doi.org/10.1038/leu.2016.143.

[31] Roider HG, Manke T, O'Keeffe S, Vingron M, Haas SA. PASTAA: identifying transcription factors associated with sets of co-regulated genes. Bioinformatics. 2009;25(4):435–442. Research Support, Non-U.S. Gov't. https://doi.org/10.1093/bioinformatics/btn627.

[32] Litmeyer AS, Konukiewitz B, Kasajima A, Foersch S, Schicktanz F, Schmitt M, et al. High expression of insulinoma-associated protein 1 (INSM1) distinguishes colorectal mixed and pure neuroendocrine carcinomas from conventional adenocarcinomas with diffuse expression of synaptophysin. Journal of Pathology: Clinical Research. 2023;9(6):498–509. https://doi.org/10.1002/cjp2.339.

[33] Luo Y, Xie C, Brocker CN, Fan J, Wu X, Feng L, et al. Intestinal PPARα Protects Against Colon Carcinogenesis via Regulation of Methyltransferases DNMT1 and PRMT6. Gastroenterology. 2019;157(3):744–759.e4. https://doi.org/10.1053/j.gastro.2019.05.057.
